# The Anti-activator QslA Negatively Regulates Phenazine-1-Carboxylic Acid Biosynthesis by Interacting With the Quorum Sensing Regulator MvfR in the Rhizobacterium *Pseudomonas aeruginosa* Strain PA1201

**DOI:** 10.3389/fmicb.2018.01584

**Published:** 2018-07-25

**Authors:** Yun-Ling Fang, Bo Chen, Lian Zhou, Zi-Jing Jin, Shuang Sun, Ya-Wen He

**Affiliations:** State Key Laboratory of Microbial Metabolism, Joint International Research Laboratory of Metabolic and Developmental Sciences, School of Life Sciences and Biotechnology, Shanghai Jiao Tong University, Shanghai, China

**Keywords:** *Pseudomonas aeruginosa*, QslA, phenazine-1-carboxylic acid, quorum sensing, MvfR

## Abstract

Two almost identical gene clusters (*phz1* and *phz2*) are responsible for phenazine-1-carboxylic acid (PCA) production in *Pseudomonas aeruginosa* (*P*. *aeruginosa*) strain MSH (derived from strain PA1201). Here, we showed that the anti-activator QslA negatively regulated PCA biosynthesis and *phz1* expression in strain PA1201 but had little effect on *phz2* expression. This downregulation was mediated by a 56-bp region within the 5′-untranslated region (5′-UTR) of the *phz1* promoter and was independent of LasR and RsaL signaling. QslA also negatively regulated *Pseudomonas* quinolone signal (PQS) production. Indeed, QslA controlled the PQS threshold concentration needed for PQS-dependent PCA biosynthesis. The quorum sensing regulator MvfR was required for the QslA-dependent inhibition of PCA production. We identified a direct protein–protein interaction between QslA and MvfR. The ligand-binding domain of MvfR (residues 123–306) was involved in this interaction. Our results suggested that MvfR bound directly to the promoter of the *phz1* cluster. QslA interaction with MvfR prevented the binding of MvfR to the *phz1* promoter regions. Thus, this study depicted a new mechanism by which QslA controls PCA and PQS biosynthesis in *P*. *aeruginosa*.

## Introduction

Phenazines are nitrogen-containing heterocyclic-ring compounds produced by diverse bacterial species (Pierson and Pierson, 2010; Shanmugaiah et al., 2010). In the clinically isolated *Pseudomonas aeruginosa* (*P*. *aeruginosa*), phenazine pyocyanin (PYO) has long been recognized as an important virulence factor required for cystic fibrosis lung infections (Lau et al., 2004; Rada and Leto, 2013). Rather than being “secondary metabolites,” phenazines were also shown to act as redox-active signaling molecules to control gene expression and community behavior in various bacterial species (Dietrich et al., 2008). Phenazine-1-carboxylic acid (PCA) and phenazine-1-carboxamide (PCN) produced by many rhizosphere pseudomonads exhibit wide-spectrum antimicrobial activity and play important roles in the biological control of plant pathogens (Chin-A-Woeng et al., 2000; Hu et al., 2005; Pierson and Pierson, 2010).

Due to the concerns that have surrounded the use of chemical pesticides since the 1980s, several studies have investigated the applications of these phenazines for the control of fungal diseases in plants (Thomashow et al., 1990; Tambong and Höfte, 2001; Dharni et al., 2012). Shenqinmycin, a new “green” biopesticide with PCA as the main active ingredient, has recently been registered in China to prevent at least nine plant diseases (Xu, 2013; Sun et al., 2017). The PCA used in such anti-fungal applications is produced by catalytic fermentation with the engineered rhizosphere strain M18 (Hu et al., 2005). To improve PCA yield in this and other bacterial strains through genetic engineering and synthetic biology, it is vital to understand the biosynthetic pathways and regulatory networks associated with PCA production.

*Pseudomonas aeruginosa* is ubiquitous in nature and utilizes a striking variety of organic compounds for the production of diverse phenazines (Jimenez et al., 2012; Lee and Zhang, 2015). All *P*. *aeruginosa* strains include two highly conserved gene clusters: *phzA_1_B_1_C_1_D_1_E_1_F_1_G_1_* (abbreviated as *phz1*) and *phzA_2_B_2_C_2_D_2_E_2_F_2_G_2_* (abbreviated as *phz2*). Both gene clusters have different promoters and flanking genes, and collectively contribute to phenazine biosynthesis in *P. aeruginosa* (Mentel et al., 2009; Li et al., 2011; Recinos et al., 2012; Cui et al., 2016; Sun et al., 2016). Phenazine biosynthesis in pseudomonads is controlled by a complex regulatory network comprised of quorum sensing (QS), two-component systems, small noncoding RNAs, and various specific and global transcriptional regulators (Wagner et al., 2003; Williams and Cámara, 2009; Sonnleitner et al., 2011; Balasubramanian et al., 2013; Sakhtah et al., 2013; Zhao et al., 2016). *P. aeruginosa* strain PA1201 is a newly identified rhizobacterium that produces high levels of PCA and PCN (Zhou et al., 2016). Using the PA1201-derived strain MSH strain, we recently investigated the effects of three QS systems on PCA biosynthesis and found that the QS systems RhlI/RhlR and *Pseudomonas* quinolone signal (PQS)/MvfR were essential for PCA biosynthesis, but the effect of the LasI/LasR QS system of PCA biosynthesis varied (Sun et al., 2016). The transcriptional factor RsaL negatively regulated *phz1*-dependent PCA biosynthesis but positively regulated *phz2*-dependent PCA biosynthesis (Sun et al., 2017). Indeed, RsaL inhibits *phz1* more strongly than it promotes *phz2* (Sun et al., 2017).

QslA is a 104-amino acid regulatory protein without a typical helix-turn-helix DNA-binding domain (Seet and Zhang, 2011). QslA was first identified as a novel anti-activator that determined the QS signal concentration threshold in *P. aeruginosa* PAO1 (Seet and Zhang, 2011). The null mutation of *qslA* increased acyl homoserine lactone-dependent QS and PQS signaling, the expression of QS-dependent genes, and the production of a range of virulence factors (Seet and Zhang, 2011). QslA also interacts with the LasR ligand-binding domain, disrupting LasR dimerization, and thereby preventing LasR from binding to its target DNA (Fan et al., 2013). The deletion of *qslA* and any other anti-activator-encoding genes resulted in the early activation of *lasB*, and a noticeable increase in *lasB* gene expression. Deletion of *qslA* in strain PAO1 significantly increased the biosynthesis of PYO (Seet and Zhang, 2011). However, the detailed mechanisms underlying this increased production remain unclear. Here, we investigated the role of QslA in PCA biosynthesis and PQS signaling in the PA1201-derived MSH strain. We also aimed to elucidate the mechanisms underlying this role.

## Materials and Methods

### Bacterial Strains, Plasmids, Primers, and Growth Conditions

All bacterial strains, plasmids, and primers used are listed in Supplementary Tables S1, S2, and S3. *E. coli* was grown in LB medium at 37°C. PA1201 and its derivatives were grown at 28°C in pigment-promoting medium (PPM) medium (following Levitch and Stadtman, 1964). When required, 20 μg/mL X-Gal (5-bromo-4-chloro-3-indolyl-β-D-galactopyranoside) was used for blue/white colony screening. Antibiotics at the following concentrations were used for *P. aeruginosa* PA1201 and its derivative strains: 50 μg/mL kanamycin, 100 μg/mL spectinomycin, and 100 μg/mL tetracycline. Antibiotics at the following concentrations were used for *E. coli*: 50 μg/mL kanamycin, 10 μg/mL gentamycin, 100 μg/mL tetracycline, and 100 μg/mL carbenicillin.

### In Frame Deletion and Complementation of qslA in *P. aeruginosa* PA1201

The method for marker-less gene deletion was previously described (He et al., 2006). The generated marker-less mutants were verified with colony polymerase chain reactions (PCR) and by subsequent DNA sequencing. For complementation, the coding and promoter regions of *qslA* were amplified and cloned into the plasmid pBBR1MCS (Kovach et al., 1995). The construct plasmid was introduced into PA1201 by conjugal mating.

### Extraction and Quantification of PCA Production

PCA was extracted with chloroform and quantified with high performance liquid chromatography (HPLC), as previously described (Jin et al., 2015). A total of 2 μL of extracts were loaded into a HPLC (1260 Infinity; Agilent Technologies) with a C18 reversed-phase column (XDB-C18 Eclipse; Agilent; 5 μm; 4.6 mm^2^ × 12.5 mm^2^). The sample was eluted with 5 mM ammonium acetate–acetonitrile (40:60 v/v).

### Construction of Reporter Strains and Measurement of (-Galactosidase Activity

The procedure for generating the promoter-*lacZ* fusion proteins was previously described by Sun et al. (2016). Briefly, ∼500 bp promoter regions and ∼30 bp coding sequences from the target genes (*phz1, phz2*, and *pqsA*) were individually PCR-amplified using the primer pairs listed in Supplementary Table S3, and cloned into mini-CTX-*lacZ* vectors (Becher and Schweizer, 2000). These constructs, verified by DNA sequencing, were introduced into the chromosomes of PA1201-derived strains by electroporation.

*P. aeruginosa* PA1201 and its derived strains carrying different fusion plasmids were cultured at 28°C with 200 rpm shaking in a 250-mL Erlenmeyer flask containing 50 mL PPM medium. Samples were collected at different time points, and β-galactosidase activity was measured as previously described (Sambrook and Russell, 2001). All experiments were performed in triplicate.

### Extraction and Quantification of PQS and HHQ in Cell Cultures With Ultra-HPLC-Time of flight (TOF)-Mass Spectrometry (UPLC-TOF-MS)

The production levels of HHQ and PQS in PA1201 and mutant strains were detected and analyzed, as previously described (Sun et al., 2016). Briefly, 540 μL of culture were collected and adjusted to pH 4.0 with 60 μL of 6 M HCl. This mixture was extracted with an equal volume of ethyl acetate. 100 μL aliquots of the ethyl acetate extracts were collected for evaporation at 42°C. The dried pellet was re-dissolved in 500 μL methanol. A 10 μL aliquot of the extracted sample was then injected into the ultra-performance liquid chromatography-mass spectrometry apparatus (UPLC1290-TOF-MS6230; Agilent). Zorbax XDB C18 reverse-phase column (Agilent; 5 μm; 4.6 mm × 150 mm) was eluted with gradient ammonium acetate containing 0.5% acetic acid, and with water containing 0.5% acetic acid, at 0.4 mL/min. The mass spectrometry analysis was performed in positive mode using a 100–1700 m/z scanning range. The concentration of QS molecules was quantified based on the peak area (A) of the specific extracted ion chromatogram in the total ion chromatogram according to the following formula: PQS (μM) = 3 × 10^-6^A + 0.4728 (R^2^ = 0.966). HHQ (μM) = 2 × 10^-7^A + 0.934 (R^2^ = 0.9826). This equation was derived from a dose–peak area plot using standard PQS and HHQ.

### Yeast Two-Hybrid Assays

Yeast two-hybrid experiment was performed using Matchmaker Gold Yeast System (Clontech) following the manufacturer’s instructions. Briefly, DNA fragments encoding QslA and different domains of MvfR were PCR amplified with primers listed in Supplementary Table S3. The resulting *qslA* gene was cloned in the prey vector pGADT7. The coding sequence of MvfR and its divided fragments were fused in-frame with the GAL4 DNA-binding domain of the bait vector pGBKT7. Each bait/prey pair was co-transformed into the yeast strain AH109.

AH109 cells carrying the transformed plasmid were grown for 3 days at 30°C on agar plates with all essential amino acids except for tryptophan, leucine, and histidine (SD-Leu-Trp-His), and adenine (SD-Leu-Trp-His-Ade). The transformants co-transformed with plasmids pGBKT7-53 and pGADT7-T (Clontech) were used as positive controls (Iwabuchi et al., 1993; Li and Fields, 1993). The strength of the protein interactions was judged by the growth of the colony on selective media following the manufacturer’s instructions.

### QslA and MvfR Protein Expression and Purification

The coding region of QslA was PCR amplified (primers are listed in Supplementary Table S3) and cloned into the expression vector pET28a (Merck, Germany). The resulting plasmid was introduced into *E. coli* strain BL21(DE3) for heterologous protein expression. Strain BL21(DE3) transformed with pET28a-*qslA* was grown in 2 L of LB medium and incubated with 0.1 mM of isopropyl-β-thiogalactopyranoside (IPTG) overnight at 16°C, with 180 rpm shaking. The collected cell pellet was resuspended in 50 mL of equilibration buffer (20 mM sodium phosphate, 300 mM sodium chloride with 10 mM imidazole, pH 7.4) and lysed with sonication. The recombinant protein was purified from the soluble cellular fraction with Ni^2+^-affinity chromatography using His-Pur Ni-NTA Resin (Pierce Biotechnology), following the manufacturer’s instructions. Protein purity was determined with sodium dodecyl sulfate-polyacrylamide gel electrophoresis.

To purify MvfR-maltose-binding protein (MBP) fusion protein, the *mvfR* coding region was cloned into pMAL-c2x (New England Biolabs Inc.). BL21 strains carrying pMAL-c2x-*mvfR* were grown in LB medium to OD_600_ = 0.45 before being induced with 0.1 mM IPTG overnight at 16°C with 180 rpm shaking. The collected cell pellet was resuspended in a solution of 25 mM Tris–HCl, 300 mM NaCl, and 2 mM EDTA (pH 8.0), and lysed with sonication. The MvfR-MBP fusion proteins were purified using amylose affinity columns (New England Biolabs Inc) and eluted with buffer containing 25 mM Tris–HCl, 300 mM NaCl, 2 mM EDTA, and 20 mM maltose (pH 8.0).

### Electrophoretic Mobility Shift Assay (EMSA)

The interactions of MvfR with its putative binding sites on the *phz1, phz2*, and *pqsA* promoters were analyzed as previously described by (Hoot et al., 2010) with minor modifications. Briefly, the probe for the corresponding region was amplified using a Cy5-labeled sense primer, the antisense primer (Supplementary Table S3), and the genomic DNA of *P. aeruginosa* PA1201 as the template. Different concentrations of the purified MvfR protein were incubated with 10 ng Cy5-labeled PCR product in a 20 μL volume containing 4 mM Tris–HCl (pH 8), 4 mM MgCl_2_, 5% glycerol, 40 nM NaCl, and 0.5 mg salmon sperm DNA for 20 min. For competitive binding, we used unlabeled oligonucleotides in excess as needed. Samples were separated on 4% polyacrylamide gels and the DNA-protein complexes were visualized using a molecular imager (Typhoon Trio Plus; GE Health Sciences).

### Far-UV Circular Dichroism Measurements

Circular dichroism measurements were carried out on a Jasco J810 spectropolarimeter equipped with peltier temperature controller system (Jasco Co., Ltd., Tokyo, Japan). The MvfR and MBP spectra were recorded in a 1 mm path length quartz cuvette in phosphate buffer (10 mM K_2_HPO_4_ and 10 mM KH_2_PO_4_; pH 7.4), with or without QslA. Spectra were averaged over three scans at a scan speed of 100 nm/min. Protein spectra between 190 and 250 nm were collected. All data were corrected for the baseline contribution of the buffer (Greenfield and Fowler, 2002). The spectra data were analyzed using Dichroweb^1^ with the non-constrained multilinear regression method.

### Chromatin Immunoprecipitation (ChIP) Assay

*Pseudomonas aeruginosa* strains were grown in liquid PPM medium to an OD_600_ = ∼2. Formaldehyde was added to make a final concentration of 1%. The culture was incubated with shaking at 28°C for 15 min. To quench the cross-linking reaction, glycine was added to make a final concentration of 250 mM, followed by a 15 min incubation at room temperature. Cell pellets were collected and washed twice with 1 × PBS buffer and were then re-suspended in lysis buffer (20 mM Tris–HCl, 50 mM KCl, 0.5 mM DTT, 10% glycerol, and 5 mM protein inhibitor, pH 7.9). Chromosomal DNA was sheared by sonication to an average size of 2–5 Kb, following the manufacturer’s instructions (Sonics Uibracell, United States) as output watts of 20 kW for 15 min, amplitude of 40% and pulse durations of 3 s ON and 6 s OFF. After the removal of cell debris by centrifugation, we used 50 μL of each sample as an input control. The immunoprecipitation reaction was initiated by the addition of anti-MBP magnetic beads (Cat # E8037S; New England Biolabs, Inc.), to each of the remaining samples. After incubation at 4°C overnight, beads were pelleted and washed three times with 0.1 M NaPhosphate buffer (pH 8.0). Beads were resuspended in elution buffer (50 mM Tris–HCl (pH 8), 10 mM EDTA (pH 8), and 1% SDS), and immunoprecipitated complexes were removed from the beads with incubation at 65°C for 15 min. The recovered supernatants were incubated at 65°C overnight to reverse cross-linkages.

### Quantitative Real-Time PCR Analysis

Quantification of gene expression and melting curve analysis were performed on a Bio-Rad Mastercycler ep Realplex 4S system (Eppendorf) using SYBR Premix Ex Taq (Takara) and the following program: 95°C for 10 min; followed by 40 cycles of 95°C for 15 s, 58°C for 20 s, 72°C for 20 s, and 68°C for 5 s followed by a plate read; with a final step of 95°C for 10 s followed by a melt curve analysis. The qRT-PCR experiments were performed in triplicates. Oligonucleotide primers used for these reactions are listed in Supplementary Table S3.

### Statistical Analysis

In this study, each experiment was independently repeated at least three times, using triplicate parallel samples within each experiment. Unless stated otherwise, each value represents the mean and SD of three replicates. Student’s *t-*test in GraphPad Pris 5 was used to evaluate the significance of difference.

### Biological Biosafety and Security

This study was carried out in accordance with the principles of the Basel Declaration and recommendations of China Biological Safety and Biosecurity Manual. The protocol was approved by the Biological Safety and Biosecurity Committee of Shanghai Jiao Tong University.

## Results

### Genetic Diversity at the qslA Loci in *P. aeruginosa*

In *P. aeruginosa* PAO1, *qslA* (PA1244) encodes a 104-amino acid protein (Seet and Zhang, 2011). Comparative genomic analysis indicated that *qslA* was present in all *P*. *aeruginosa* strains available in the *Pseudomonas* Genome Database.^2^ However, the regions downstream of *qslA* were not conserved across *P. aeruginosa* strains. Based on these heterogeneities, we classified all available *P. aeruginosa* into three groups. Group I is represented by the strain PAO1, including strains PAO1, LESB58, UCBPP-PA14, DK2, and most of the other *P*. *aeruginosa* strains. In group I strains, the genes PA1243, encoding a sensor/response regulator hybrid protein, and PA1242, encoding a hypothetical protein, were located directly downstream of *qslA* (Supplementary Figure S1A). The GC content of *qslA* was 63.74%, 6.06% lower than the two neighboring genes (69.80%; Supplementary Figure S1A).

Group II is represented by the *P. aeruginosa* strain M18, including strains M18, PA1201, PA96, and PcyII-10. Two new genes (PAM18_3788 and PAM18_3789) were inserted between *qslA* and the genes homologous to PA1242 and PA1243 in strain PAO1 (Supplementary Figure S1A). PAM18_3788 and PAM18_3789 both encode hypothetical proteins. Deletion of PAM18_3788 and PAM18_3789 had little effect on bacterial growth and PCA biosynthesis (Supplementary Figure S2). The GC contents of PAM18_3788 and PAM18_3789 were 53.71 and 44.63%, respectively. These levels were much lower than the 69.76% GC content of the neighboring genes (Supplementary Figure S1A).

Group III is represented by the *P. aeruginosa* strain RP73, including strains RP73, F63912, and DHS01. In this group, an 8.367-Kb region was inserted between *qslA* and the genes homologous to PA1243 and PA1242 in PAO1 (Supplementary Figure S1A). The GC content of the 8.367-Kb region was 65.24%, which is lower than 69.75% GC content of the neighboring genes. The 8.367-Kb region contained eight genes encoding CRISPR-associated enzymes and proteins (Supplementary Figures S1A,B).

### QslA Negatively Regulates PCA Biosynthesis in PA1201

**FIGURE 1:**
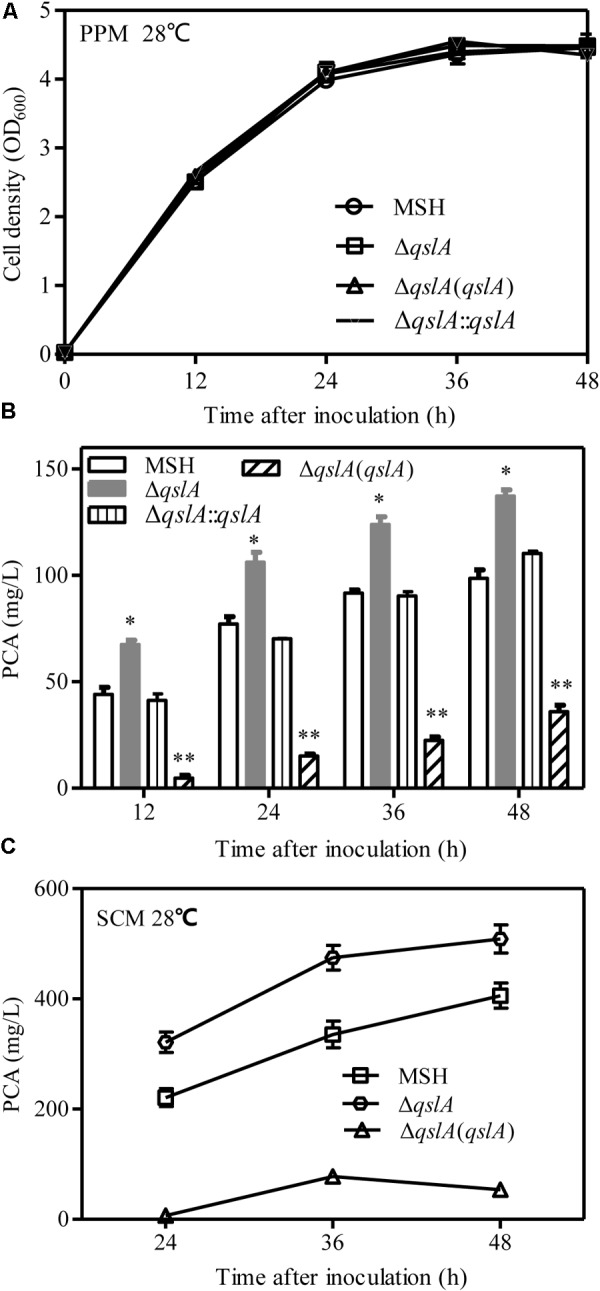
QslA is a negative regulator of PCA biosynthesis. **(A)** Bacterial growth in PPM. **(B)** PCA production by PA1201-derived strains in PPM. **(C)** PCA production by PA1201-derived strains in SCM medium. Three replicates were performed for each strain; and error bars indicate SDs. Statistical significance with respect to the MSH strain is indicated with asterisks (*P* < 0.05).

To investigate the role of QslA in PCA biosynthesis, *qslA* was in frame deleted from the PA1201-derived strain MSH, which has deletions of the *phzM, phzS*, and *phzH* genes that result in the production of PCA as the sole phenazine (Jin et al., 2015). Deletion of *qslA* did not significantly affect bacterial growth in PPM (**Figure 1A**). The Δ*qslA* strain produced significantly more PCA than did the unaltered MSH strain at all-time points tested (*P* < 0.05 at 12, 24, 36, and 48 h after inoculation; **Figure 1B**), an increase of up to 1.6 times the amount produced by strain MSH. Integration of a single copy of *qslA* in the strain Δ*qslA* restored PCA biosynthesis to MSH levels. Complementation of the Δ*qslA* mutant strain with a pBBR1MCS-derived multicopy plasmid reduced PCA biosynthesis significantly compared to strain MSH (**Figure 1B**). Similar trends in PCA production were observed when MSH, Δ*qslA*, and Δ*qslA*(*qslA*) were grown in the SCM medium (Zhou et al., 2010) containing soybean meal, corn steep liquor, and ethanol as nitrogen and carbon sources for the industrial production of PCA for the synthesis of the biopesticide shenqinmycin (**Figure 1C**).

### QslA Negatively Regulates phz1 Expression and phz1-Dependent PCA Biosynthesis

**FIGURE 2:**
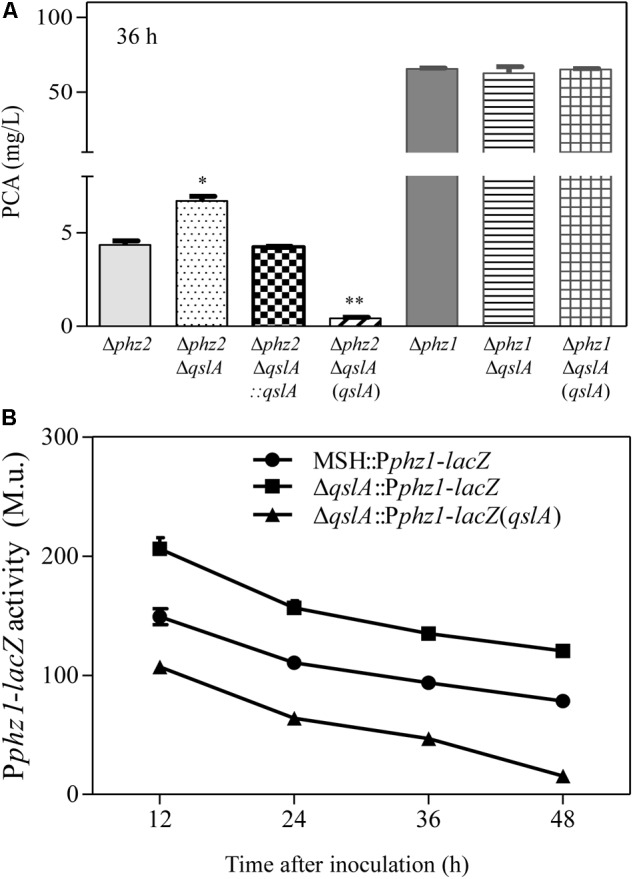
QslA negatively regulates *phz1* expression and *phz1*-dependent PCA biosynthesis. **(A)** PCA production by the Δ*phz1*- and Δ*phz2*-derived strains 36 h after inoculation. **(B)** The relative expression of *phz1* as compared to β-galactosidase activity. Each bar represents the mean of three independent experiments; error bars indicate SDs. Statistical significance with respect to the respective parental strain is indicated by one or two asterisks (*P* < 0.05).

PA1201 contains two almost identical operons, *phz1* and *phz2*, both of which are involved in PCA synthesis (Sun et al., 2016). In Δ*phz1*, PCA biosynthesis depends on the *phz2* operon (*phz2*-dependent PCA biosynthesis), while in Δ*phz2*, PCA biosynthesis depends on the *phz1* operon (*phz1*-dependent PCA biosynthesis; Sun et al., 2017). To determine how QslA inhibits PCA biosynthesis, we generated two strains, *Δphz1*Δ*qslA* or Δ*phz2*Δ*qslA*. When the strains were grown in PPM medium, *qslA* deletion had no effects on growth (Supplementary Figure S3A). In this medium, PCA production 36 h after inoculation in strain Δ*phz2*Δ*qslA* (6.7 mg/L) was significantly higher than in strain Δ*phz2* (4.3 mg/L; *P* < 0.05), while PCA production in strain Δ*phz2*Δ*qslA*(*qslA*) was significantly lower (0.4 mg/L; *P* < 0.05). In contrast, PCA production in strain Δ*phz1*Δ*qslA* 36 h after inoculation (62.5 mg/L) was not significantly different from that of Δ*phz1* (65.4 mg/L) or Δ*phz1*Δ*qslA*(*qslA*) (65.0 mg/L; **Figure 2A**). These results indicated that QslA negatively regulated *phz1*-dependent PCA biosynthesis, but not *phz2-*dependent PCA biosynthesis.

To further clarify the regulatory effects of QslA on *phz* clusters, *qslA* was deleted or overexpressed in two previously described reporter strains that monitor the activity levels of *phz1* and *phz2* (Δ*phz2*::P*phz1*-*lacZ* and Δ*phz1*::P*phz2*-*lacZ*, respectively; Sun et al., 2016). We then compared β-galactosidase activity of each of the resulting strains Δ*qslA*Δ*phz2*::P*phz1*-*lacZ*, Δ*qslA*Δ*phz2*::P*phz1*-*lacZ*(*qslA*), Δ*qslA*Δ*phz1*::P*phz2*-*lacZ*, and Δ*qslA*Δ*phz1*::P*phz2*-*lacZ*(*qslA*) with that of their respective parental strains. When grown in PPM medium, the β-galactosidase activity of Δ*phz2*Δ*qslA*::P*phz1*-*lacZ* was significantly higher than that of Δ*phz2*::P*phz1*-*lacZ* at all-time points tested (*P* < 0.05 at 12, 24, 36, and 48 h after inoculation), while the β-galactosidase activity of the strain Δ*qslA*Δ*phz2*::P*phz1*-*lacZ* overexpressing *qslA* was significantly lower (*P* < 0.05 at 12, 24, 36, and 48 h after inoculation; **Figure 2B**). In contrast, neither the deletion nor the overexpression of *qslA* in the reporter strain Δ*phz1*::P*phz2*-*lacZ* had a significant effect on β-galactosidase activity at any stage (Supplementary Figure S3C). These results suggested that QslA negatively regulated *phz1* transcriptional activity independently of *phz2*.

### QslA Inhibition of PCA Biosynthesis Was Mediated by a 56-bp Region Within the 5′-UTR of the phz1 Promoter

**FIGURE 3:**
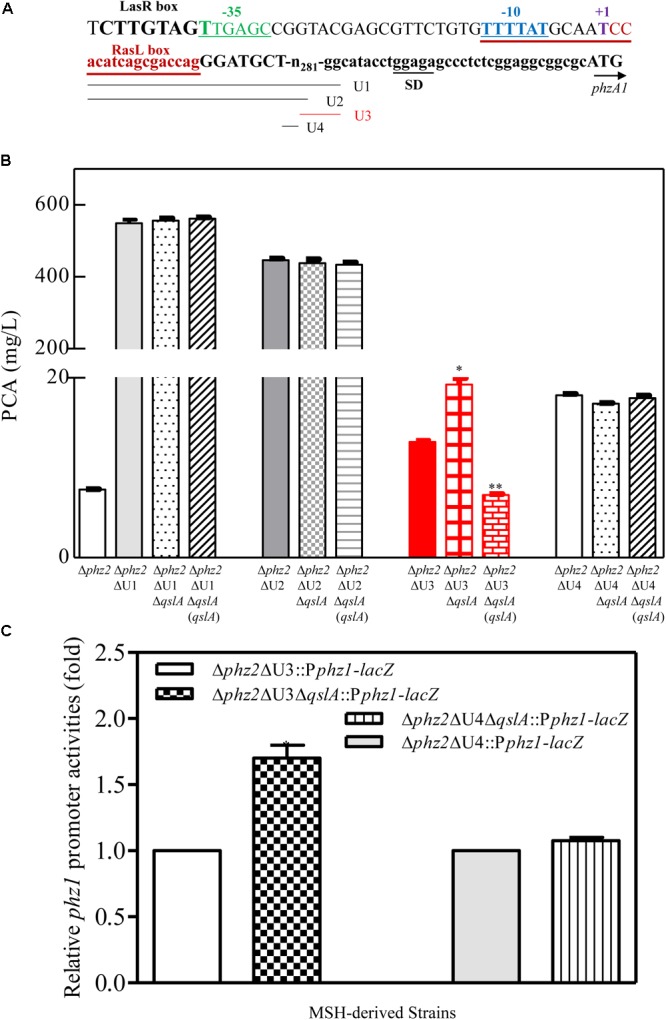
QslA negatively regulates PCA biosynthesis via the U4 region of the *phz1* promoter. **(A)** Schematic diagram of the promoter region of the *phz1* gene cluster. The labels U1–U4 indicate the regions deleted in this study. The LasR and RsaL boxes, transcription initiation site (+1), the –10/–35 sequences, and the Shine-Dalgarno sequence are underlined and labeled accordingly. **(B)** PCA production by the Δ*phz2-*derived strains. **(C)** The relative *phz1* promoter activity indicated by β-galactosidase activity. Each bar represents the mean of duplicate assays from at least three independent experiments; error bars indicate SDs. Statistical significance with respect to the wild-type strain is indicated by one or two asterisks (*P* < 0.05).

Previously, Li et al. (2011) reported that efficient expression of the *phz1* cluster in M18 was blocked by its 5′-UTR during a post-transcriptional event. The same 5′-UTR was identified within the *phz1* promoter region, but not within the *phz2* promoter region in strain MSH; deletion of this 5′-UTR led to a significant increase in PCA production (Jin et al., 2015; Sun et al., 2017). In this study, our results indicated that deletion or overexpression of *qslA* in the 5′-UTR-deletion mutant Δ*phz2*ΔU1 had little effect on PCA biosynthesis (**Figure 3B**), suggesting that QslA indirectly regulated *phz1* expression via the 5′-UTR.

To further specify the QslA-target region within the *phz1* promoter, the 5′-UTR (U1; 305 bp) was subdivided into three subregions: U2 (250 bp), U3 (227 bp), and U4 (56 bp) (**Figure 3A**). Each subregion was individually deleted in the strains Δ*phz2* and Δ*phz2*Δ*qslA*. Neither the deletion of *qslA* in strains Δ*phz2*ΔU2 and Δ*phz2*ΔU4 nor the overexpression of *qslA* in strains Δ*phz2*ΔU2Δ*qslA* and Δ*phz2*ΔU4Δ*qslA* affected PCA biosynthesis (24 h post-inoculation; **Figure 3B**). However, the deletion of *qslA* in strain Δ*phz2*ΔU3 significantly increased PCA biosynthesis (*P* < 0.05), while the overexpression of *qslA* in strain Δ*phz2*Δ*qslA*ΔU3 significantly decreased PCA biosynthesis (*P* < 0.05; **Figure 3B**). Furthermore, we generated two pairs of *lacZ*-based reporter strains to compare the effects of U3 and U4 regions on *phz1* promoter activity: (1) Δ*phz2*ΔU3::P*phz1*-*lacZ* and Δ*phz2*Δ*qslA*ΔU3::P*phz1*-*lacZ*; (2) Δ*phz2*ΔU4::P*phz1*-*lacZ* and Δ*phz2*Δ*qslA*ΔU4::P*phz1*-*lacZ*. Analysis of the relative β-galactosidase activities at 24 h after inoculation showed that deletion of U3 significantly increased *phz1* promoter activity, whereas deletion of U4 had little effect on *phz1* promoter activity (**Figure 3C**). The 56-bp U4 region was only identified in the *phz1* promoter, but not in the *phz2* promoter (Li et al., 2011; Jin et al., 2015). These findings demonstrated that QslA negatively regulated *phz1* expression via the 56-bp U4 region within the *phz1* promoter.

### QslA Negatively Regulated PCA Biosynthesis Independent of LasR and RsaL Signaling

**FIGURE 4:**
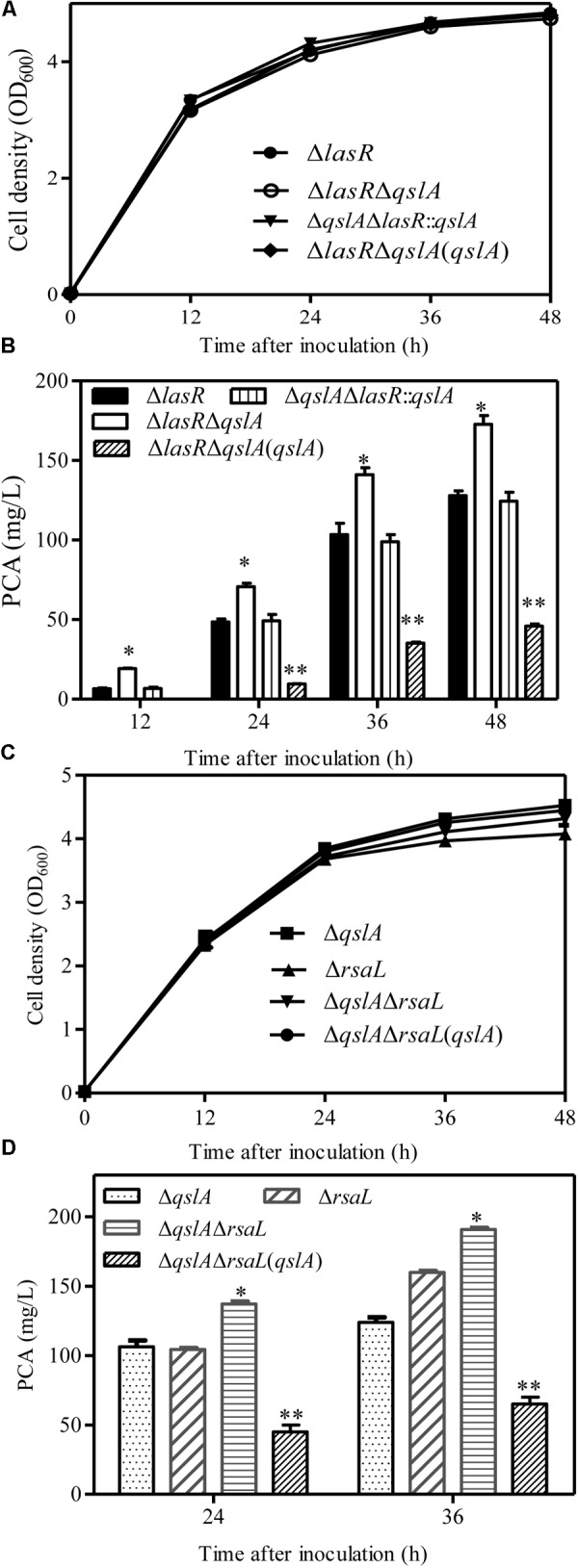
QslA regulates PCA biosynthesis independent of the LasR and RsaL signaling systems. **(A)** Growth of the Δ*lasR-* and Δ*lasR*-derived bacterial strains in PPM. **(B)** PCA production by the Δ*lasR-* and Δ*lasR*-derived strains. **(C)** Growth of the Δ*rsaL* and Δ*rsaL*Δ*qslA* bacterial strains in PPM. **(D)** PCA production by the Δ*rsaL* and Δ*rsaL*Δ*qslA* strains in PPM. Three replicates were performed for each strain; error bars indicate SDs. Statistical significance with respect to each parental strain is indicated by one or two asterisks (*P* < 0.05).

In *P*. *aeruginosa* strain PAO1, QslA has been reported to interact with the QS regulator LasR to negatively regulate the expression of QS-related genes (Seet and Zhang, 2011; Fan et al., 2013). Here, a weak interaction between QslA and LasR was observed in a yeast two-hybrid analysis: the AH109 strain expressing QslA and LasR grew on the SD-Leu-Trp-His-selective plate, but not on the SD-Leu-Trp-His-Ade-selective plate (Supplementary Figure S4A). To further verify that QslA regulates PCA biosynthesis via LasR in PA1201, we generated three strains: *lasR* deletion mutant carrying pBBR vector (Δ*lasR*), *lasR* and *qslA* double deletion mutant carrying pBBR vector (Δ*qslA*Δ*lasR*), and the strain Δ*qslA*Δ*lasR* overexpressing *qslA* via pBBR vector. The three strains grew at approximately the same rate in PPM medium (**Figure 4A**). Deletion of *qslA* in strain Δ*lasR* significantly increased PCA production as compared to that of Δ*lasR*, while overexpression of *qslA* in strain Δ*qslA*Δ*lasR* significantly decreased PCA production as compared to that of Δ*lasR* (**Figure 4B**). These results suggested that QslA negatively regulated PCA biosynthesis independent of LasR.

Previous studies have shown that the 90-amino acid regulator RsaL binds the *phz1* promoter directly to negatively regulate PCA biosynthesis in the strain MSH (Sun et al., 2017). To verify the involvement of RsaL in QslA signaling, we generated the *qslA* and *rasL* double deletion mutant Δ*qslA*Δ*rasL*. We found that the deletion of *qslA* in strain Δ*rasL* further increased PCA production, while the overexpression of *qslA* in strain Δ*qslA*Δ*rasL* significantly decreased PCA production (**Figures 4C,D**). PCA production of strain Δ*qslA*Δ*rasL* was significantly greater than that of either strain Δ*qslA* or strain Δ*rasL* (**Figures 4C,D**), suggesting that these proteins had an additive effect on PCA biosynthesis. Further yeast two-hybrid analyses revealed no direct protein–protein interactions between QslA and RsaL (Supplementary Figure S4B).

### QslA Inhibited PQS Signaling System

**FIGURE 5:**
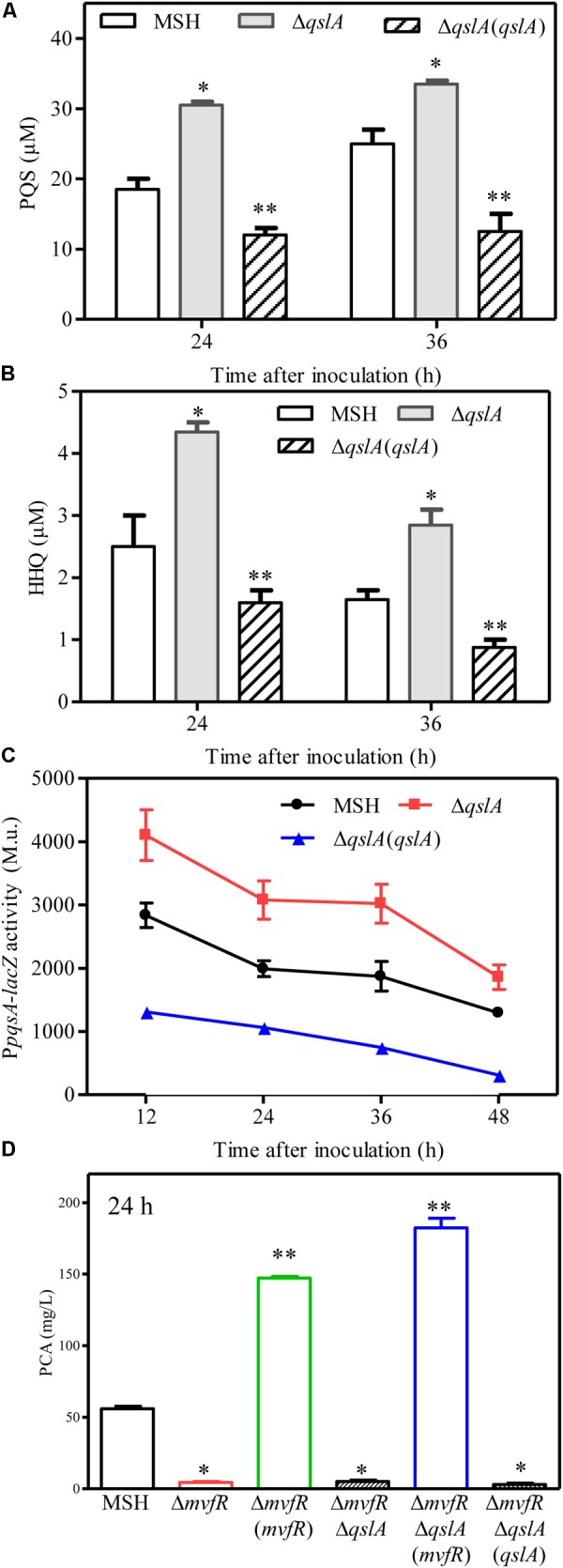
QslA negatively regulates PQS signaling. **(A)** The production of extracellular PQS by the strains Δ*qslA* and Δ*qslA*(*qslA*). **(B)** The production of extracellular HHQ by the strains Δ*qslA* and Δ*qslA*(*qslA*). **(C)** The transcriptional activity of *pqsA* relative to β-galactosidase during growth. M.u.: Miller Unit. **(D)** MvfR acts downstream of QslA to positively regulate PCA biosynthesis. Each bar represents the average of three independent experiments; error bars indicate SDs. Statistical significance with respect to strain MSH is indicated by one or two asterisks (*P* < 0.05).

HHQ and PQS are the two primary quinolone signals produced by *P*. *aeruginosa* (Wade et al., 2005). Here, we analyzed HHQ and PQS levels in *qslA* deletion and overexpression strains by UPLC-TOF-MS, as previously described (Sun et al., 2016). Our results indicated that *qslA* deletion significantly increased HHQ and PQS production as compared to the wild-type strain, while *qslA* overexpression significantly reduced HHQ and PQS production as compared to the wild-type strain (**Figures 5A,B**).

The genes *pqsABCD* and *pqsH* encode the enzymes that synthesize HHQ and PQS (Wade et al., 2005). To further investigate the effects of *qslA* on the transcriptional activity of the *pqsABCDE* operon, we constructed three MSH-derived reporter strains: MSH::P*pqsA*-*lacZ*, Δ*qslA*::P*pqsA*-*lacZ*, and the Δ*qslA*::P*pqsA*-*lacZ* strain overexpressing *qslA*, using a previously described method (Sun et al., 2016). β-galactosidase assays showed that *qslA* deletion significantly increased *pqsA* expression during growth, while *qslA* overexpression significantly decreased *pqsA* expression during growth (**Figure 5C**).

### QslA Modulated PQS Sensitivity Required for PCA Biosynthesis

**FIGURE 6:**
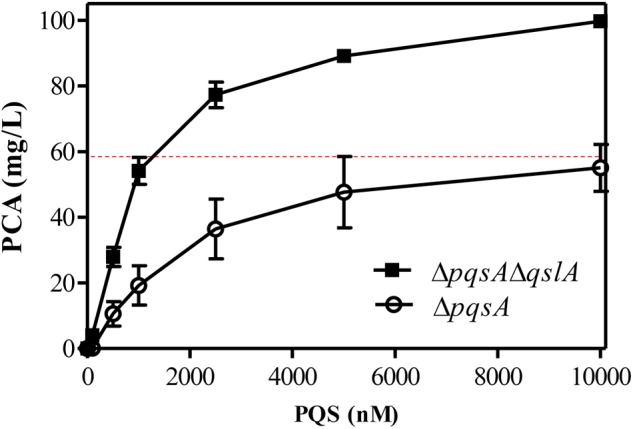
QslA controls the PQS threshold concentration needed for QS-dependent PCA biosynthesis. PCA production by the strains Δ*pqsA* and Δ*pqsA*Δ*qslA* was measured in PPM supplemented with various concentrations of PQS. The dotted line indicates the PCA production of the MSH strain (derived from PA1201) without the addition of exogenous PQS, under the same experimental conditions. Each bar represents the average of three independent experiments; error bars indicate SDs.

QslA influences the threshold concentration of 3-oxo-C12-HSL needed for QS-dependent elastase and protease induction in strain PAO1 (Seet and Zhang, 2011). The expression of the PQS signaling gene was increased in the MSH-derived *qslA* deletion mutant, and PQS-dependent PCA biosynthesis was enhanced, as compared to MSH (**Figure 5D**). This result led us to question whether bacterial populations might become more sensitive to PQS in the absence of *qslA*. To test this possibility, we generated the single deletion mutant Δ*pqsA* and the double deletion mutant Δ*pqsA*Δ*qslA.* We then determined the amount of exogenous PQS necessary for the induction of QS-dependent PCA biosynthesis in these mutants. We found that 1,000 nM PQS was required to increase the biosynthesis of PCA by Δ*pqsA*Δ*qslA* to wild-type levels; 10 times this amount of PQS required to produce wild-type levels of PCA in strain Δ*pqsA* (**Figure 6**). This suggested that, in strain PA1201, QslA controlled the PQS threshold concentration required for PCA biosynthesis.

### QslA Interacted Directly With MvfR

The PQS signal receptor MvfR regulates HHQ and PQS biosynthesis by binding the promoter of *pqsABCD* (Wade et al., 2005; Xiao et al., 2006; Maura et al., 2016). *mvfR* is essential for PCA biosynthesis in strain PA1201 (Sun et al., 2016). Here, to investigate whether MvfR was required for QslA signaling, we generated the *mvfR* and *qslA* double deletion mutant Δ*mvfR*Δ*qslA*. We found that this double mutant produced similar amounts of PCA as the strain Δ*mvfR* (**Figure 5D**). Overexpression of *mvfR* in the double mutant Δ*mvfR*Δ*qslA* fully restored PCA production levels to those produced by the overexpression of *mvfR* in strain Δ*mvfR* (**Figure 5D**). However, the overexpression of *qslA* in the double deletion mutant Δ*mvfR*Δ*qslA* had little effect on PCA biosynthesis (**Figure 5D**). These results suggested that inhibition of PCA biosynthesis by QslA depended on MvfR.

**FIGURE 7:**
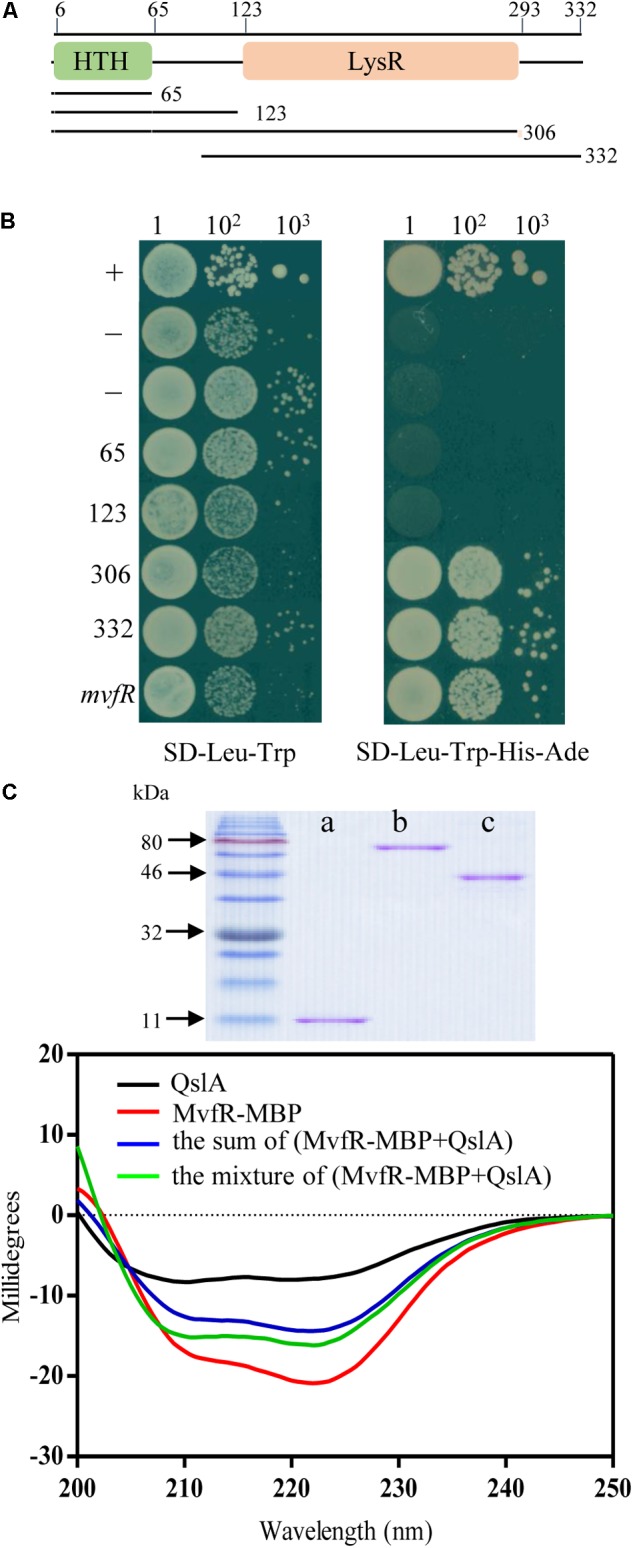
QslA interacts with MvfR. **(A)** Domain organization of MvfR. **(B)** Yeast two-hybrid assays showing the interaction between QslA and different regions of MvfR. A yeast strain carrying the vector pGADT7 was used as the negative control. A yeast strain carrying pGADT7-T and pGBKT7-53 was used as the positive control. **(C)** Purified proteins and circular dichroism spectra. CD measurements were carried out on a Jasco J810 spectropolarimeter equipped with peltier temperature controller system (Jasco Co., Ltd., Tokyo, Japan). a: QslA (11.8 kDa), b: MvfR-MBP (77.3 kDa), and c: MBP tag (40.1 kDa).

To investigate whether QslA and MvfR interact directly in strain PA1201, these genes were expressed in the yeast strain AH109 via the plasmids pGADT7 and pGBKT7. Both resulting strains grew well on the selective plates SD-Leu-Trp and SD-Leu-Trp-His-Ade (**Figure 7B**). Strains overexpressing QslA or MvfR (negative controls) failed to grow on the same selective plates (**Figure 7B**). We also investigated the interaction between QslA and MvfR using circular dichroism spectroscopy. Although we failed to express and purify the full soluble MvfR protein, the MvfR-His6 fusion protein, or the MvfR-GST fusion protein using *E. coli* strain BL21, we did obtain the soluble MvfR-MBP fusion protein expressed in *E. coli* strain BL21 using an amylose affinity column (**Figure 7C**). QslA was expressed in *E. coli* strain BL21 and purified with Ni^2+^-affinity chromatography (**Figure 7C**). QslA and MvfR-MBP had obviously different CD spectra as shown in black and red, respectively, in **Figure 7C**. Compared to the sum spectrum of QslA and MvfR-MBP (shown in blue), increases in negative ellipticity at 208 nm (α-helix), 218 nm (β-sheet), and 222 nm (α-helix) were observed in the spectra of the 1:1 MvfR-MBP mixture (**Figure 7C**). The ellipticity of the MvfR-MBP mixture spectra was not significantly different to that of the sum spectra (Supplementary Figure S5), suggesting no direct interaction between MBP and QslA.

MvfR contained an N-terminal helix-turn-helix DNA-binding domain (∼0–65 amino acids) and a C-terminal LysR substrate domain (∼123–306 amino acids; **Figure 7A**). To further investigate how QslA interacted with MvfR, *mvfR* was divided into four fragments, encoding peptides of 65, 123, 306, and 332 amino acids (**Figure 7A**). Each of these four DNA fragments was expressed in separate AH109 yeast strains via the vector pGBKT7, along with QslA, expressed via the vector pGADT7. Only strains expressing QslA plus the C-terminal 306 amino acid region or the 332 amino acid region grew well on the SD-Leu-Trp-His-Ade selective plates (**Figure 7B**). These results suggested that QslA probably interacted with MvfR in the region between the 123rd and the 306th amino acid.

### The Interaction of QslA and MvfR Affected the Binding of MvfR to the phz1 Promoter

We next investigated whether MvfR directly binds the *phz1* promoter to regulate PCA biosynthesis. We tried to investigate this interaction with an EMSA. Although we failed to obtain the soluble MvfR protein, we found that overexpression of the *mvfR*-MBP fusion gene in strain Δ*mvfR* fully restored PCA biosynthesis to wild-type level (Supplementary Figure S6). These results suggested that the MBP tag did not affect the function of MvfR-MBP inside the strain MSH. We therefore used the purified MvfR-MBP fusion protein for the EMSA assay. We used a CY5-labeled 256-bp *phz1* promoter fragment and a CY5-labeled 300-bp *pqsA* promoter fragment as probes. No band shifts were detected in the MvfR-MBP EMSA assays, or in the MvfR-MBP plus QslA EMSA assays (Supplementary Figures S7A–C). Our positive control, the purified transcriptional factor MvaU, was observed to bind to the *phz2* promoter region (Supplementary Figure S7D).

**FIGURE 8:**
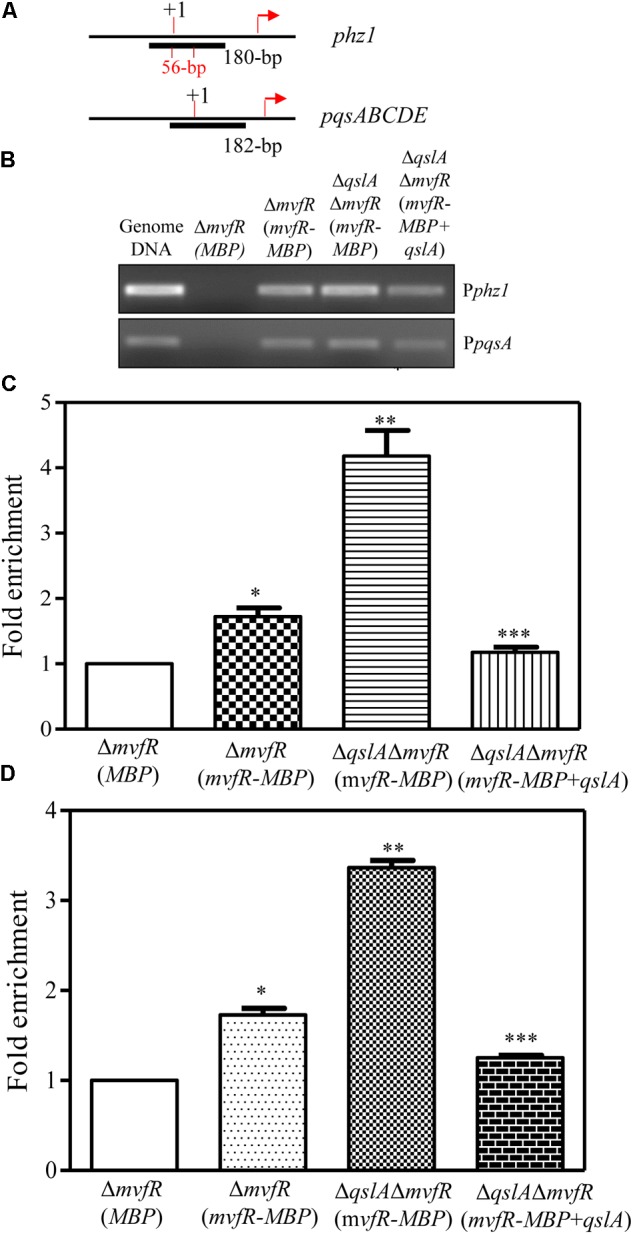
ChIP-qPCR analyses showing the *in vivo* interaction between QslA/MvfR and the *phz1* promoter region. **(A)** The ChIP fragment localized on the *phz1* and *pqsA* promoters. **(B)** The 180-bp PCR products generated using immunoprecipitated DNA as templates and the primers specific to the *phz1* promoter. Protein/DNA complexes isolated from the cell cultures were immunoprecipitated with the anti-MBP magnetic beads (Cat # E8037S; New England Biolabs, Inc.). **(C,D)** qRT-PCR analysis of the relative enrichment of the *phz1* and *pqsA* promoters in the immunoprecipitated DNA derived from the three strains. “+1” indicates the transcription initiation site and the arrows indicate the translational initiation site. Each bar represents the mean of three independent experiments; error bars indicate SDs. Statistical significance with respect to the strain Δ*mvfR*(MBP) is indicated by one, or two, or three asterisks (*P* < 0.05).

We then used ChIP assays to investigate whether MvfR-MBP binds the *phz1* promoter *in vivo*. We constructed two strains for this assay: Δ*mvfR* overexpressing MBP [referred to as Δ*mvfR*(MBP)] and Δ*mvfR* overexpressing *mvfR*-MBP [referred to as Δ*mvfR*(*mvfR*-MBP)]. We used anti-MBP magnetic beads, which contain monoclonal antibodies against MBP, to select the DNA fragments that were cross-linked with MvfR-MBP. We used ChIP-polymerase chain reactions (PCRs) to identify the recovered DNA fragments (Supplementary Figure S8 and **Figures 8A,B**). As controls for the ChIP-PCRs, we amplified total DNA and DNA recovered from a mock immunoprecipitation (**Figure 8B**). We detected a 180-bp PCR product corresponding to the *phz1* promoter and a 182-bp PCR product corresponding to the *pqsA* promoter in the anti-MBP immunoprecipitates; these products were not detected in the mock immunoprecipitate from strain Δ*mvfR*(MBP). These results suggested that MvfR directly bound the promoter regions of *phz1* and *pqsA*.

Further ChIP assays were conducted for the strains Δ*mvfR*(*mvfR*-MBP), Δ*qslA*Δ*mvfR*(*mvfR*-MBP), and Δ*qslA*Δ*mvfR*(*mvfR*-MBP) overexpressing *qslA* (**Figure 8**). Using ChIP-PCR, we identified two products corresponding to the *phz1* and *pqsA* promoter regions in all anti-MBP immunoprecipitates. Furthermore, quantitative real-time PCR analysis of the anti-MBP immnoprecipitates showed that deletion of *qslA* in strain Δ*mvfR* (*mvfR*-MBP) significantly increased the fold enrichment of the immunoprecipitated DNA of the *phz1* and *pqsA* promoters (**Figures 8C,D**). Overexpression of *qslA* in the strain Δ*qslA*Δ*mvfR*(*mvfR*-MBP) significantly reduced the fold enrichment of the immunoprecipitated DNA of the *phz1* and *pqsA* promoters (**Figures 8C,D**).

## Discussion

In this study, we presented evidence that QslA downregulated PCA biosynthesis in *P*. *aeruginosa* 1201. The null mutation of *qslA* increased *phz1* expression and PCA biosynthesis, while *in trans* expression of *qslA* almost eliminated *phz1* expression as well as PCA biosynthesis (**Figures 1, 2**). QslA regulation of PCA biosynthesis was mediated via a 56-bp region of the *phz1* promoter; this regulatory mechanism was independent of the LasR and RsaL signaling systems (**Figure 4**). We further showed that QslA negatively regulated PCA biosynthesis via the PQS signaling system, and the PQS signal receptor MvfR directly bound the *phz1* promoter. The interaction of QslA and MvfR affected the binding of MvfR to the *phz1* promoter (**Figure 8**). Thus, our results identified a new QslA-interacting protein and a novel regulatory mechanism for PCA biosynthesis in *P*. *aeruginosa*.

Previous studies of strain PAO1 have shown that QslA negatively regulates AHL and PQS signaling (Seet and Zhang, 2011). In the strain MSH, our results confirmed that QslA negatively regulated PQS and HHQ biosynthesis, as well as the expression of the PQS synthase gene cluster *pqsABCD* (**Figure 5**). QslA modulated PQS sensitivity (**Figure 6**). We further showed that the PQS signal receptor MvfR directly bound the *pqsA* promoter (**Figure 8**). The interaction of QslA with MvfR affected the binding of MvfR to the *pqsA* promoter (**Figure 8**). Taken together, these findings explained how QslA modulates the sensitivity of PQS-dependent PCA biosynthesis and suggested that QslA was a key regulator of AHL- and PQS-dependent QS systems in *P*. *aeruginosa*. Our analysis of the multiple roles of QslA provided further insight into the fine and complicated mechanisms regulating QS in *P*. *aeruginosa*. Bacteria may recruit QslA to prevent premature QS activation at low population densities by increasing the thresholds for the QS signals 3-oxo-C12 and PQS, and thus only initiating QS-related gene expression at optimal bacterial densities (Seet and Zhang, 2011).

MvfR is one of the most important QS regulators in *P*. *aeruginosa* (Wade et al., 2005). In the opportunistic human pathogenic strains PAO1 and PA14, MvfR directly regulates the expression of *phnAB* and *pqsABCDE* to control PQS signal biosynthesis (Xiao et al., 2006). MvfR also regulates the production of a range of virulence factors, such as elastase, phospholipase, and PYO (Cao et al., 2001; Déziel et al., 2005; Diggle et al., 2006). An RNA-seq analysis of the MvfR regulon identified 770 differentially expressed genes, including several LasR- and RhlR-activated QS genes (Maura et al., 2016). In the rhizosphere strain PA1201, *mvfR* was shown to be essential for PCA biosynthesis; the deletion of *mvfR* completely disrupted PCA biosynthesis (Sun et al., 2016). However, the mechanisms underlying MvfR regulation remain less well studied. One of the challenges associated with the study of MvfR is that full-length MvfR is insoluble when expressed in *E*. *coli* (Kefala et al., 2012; Ilangovan et al., 2013). This greatly limits the use of *in vitro* EMSAs and subsequent foot-printing analyses. Previous EMSA assays showing MvfR directly binding to the *pqsA* promoter were performed using total soluble proteins containing MvfR; these proteins were derived from *mvfR* overexpression in *E. coli* strain DH5α (Wade et al., 2005; Xiao et al., 2006). Here, although overexpression of the *mvfR*-MBP fusion gene in strain Δ*mvfR* fully restored PCA biosynthesis (Supplementary Figure S7), no band shifts were detected in EMSA assays using the soluble MvfR-MBP fusion proteins in the absence or presence of QslA (Supplementary Figure S7). This was likely because the MBP tag affected the binding of MvfR to the *phz1* or *pqsA* promoters *in vitro*. Nevertheless, the available data, albeit limited, could not rule out another possibility that an additional protein or co-factor is required for the binding of MvfR to *phz1* promoter in *P*. *aeruginosa*. Further tests of this possibility, and the specific identification of the MvfR binding site on the promoters of the target genes, would increase our understanding of the sophisticated regulatory mechanisms associated with the MvfR signaling network in *P*. *aeruginosa*.

QslA is an anti-activator that acts by modulating QS through molecular interaction with the QS signal receptor LasR in *P. aeruginosa* (Seet and Zhang, 2011). Crystal structural analysis of full-length QslA in complex with the LasR ligand-binding domain (LBD; amino acids 1–170) revealed that QslA binding occupies the LasR dimerization interface, therefore preventing LasR from binding to the promoter of its target gene (Fan et al., 2013). How QslA interacts with full-length LasR remains unclear. The present study showed that QslA interacted with the LBD domain of the PQS receptor MvfR to regulate PQS and PCA biosynthesis independent of LasR in the strain MSH (**Figures 4, 7**). Based on the known crystal structure of the MvfR LBD (Kefala et al., 2012; Ilangovan et al., 2013), there was little homology between MvfR LBD and LasR LBD. It is thus unclear how QslA interacts with MvfR. The soluble full-length MvfR-MBP fusion protein obtained in this study might be useful for the determination of the structure of the QslA/MvfR complex, and for further insights into the molecular interactions of this complex.

Although *qslA* is present in all *P. aeruginosa* strains, a genetic diversity in the *qslA* loci was revealed in this study (Supplementary Figure S1). Based on GC content analysis, it seems that *qslA* loci in *P. aeruginosa* strains are obtained through HGT mechanism. HGT plays an important role in the evolution, maintenance, and transmission of virulence in prokaryotes (Keen, 2012; Arber, 2014). *P. aeruginosa* is one of the most abundant organisms on earth and has been found in environments such as soil, water, humans, animals, plants, sewage, and hospitals (Jimenez et al., 2012; Lee and Zhang, 2015). Whether *qslA* loci diversity is associated with the ability to adapt to ubiquitous ecological niches deserve further investigation. In addition, QslA is a small protein, composed of four α-helices and a short two-stranded antiparallel β-sheet following the N-terminal α-helix (Fan et al., 2013). It represents a unique fold without DNA-binding activity (Fan et al., 2013). Our data and previous findings suggested that QslA was prone to interaction with LBD-containing transcriptional factors, such as LasR and MvfR (**Figure 4**; Fan et al., 2013). Since *P. aeruginosa* contains diverse LBD-containing proteins and transcriptional factors, it would be interesting to determine whether more QslA-interacting proteins exist.

## Author Contributions

Y-WH, Y-LF, LZ, and BC conceived and planned the experiments. Z-JJ carried out the LacZ assays. SS planned and carried out the deletion and reporter strains assay. Z-JJ contributed to sample preparation. LZ, Y-LF, and Y-WH contributed to the interpretation of the results. Y-WH took the lead in writing the manuscript. All authors provided critical feedback and helped shape the research, analysis, and manuscript.

## Conflict of Interest Statement

The authors declare that the research was conducted in the absence of any commercial or financial relationships that could be construed as a potential conflict of interest.

## Footnotes

1 http://dichroweb.cryst.bbk.ac.uk

2 http://www.pseudomonas.com
