# Supplementary material for: The Anti-activator QslA Negatively Regulates Phenazine-1-Carboxylic Acid Biosynthesis by Interacting With the Quorum Sensing Regulator MvfR in the Rhizobacterium Pseudomonas aeruginosa Strain PA1201
Source: Front Microbiol. 2018 Jul 25;9:1584. doi: 10.3389/fmicb.2018.01584 (PMC6068238; doi:10.3389/fmicb.2018.01584)
Supplement: Supplementary file 1 [file Presentation_1.pptx]

## Slide 1
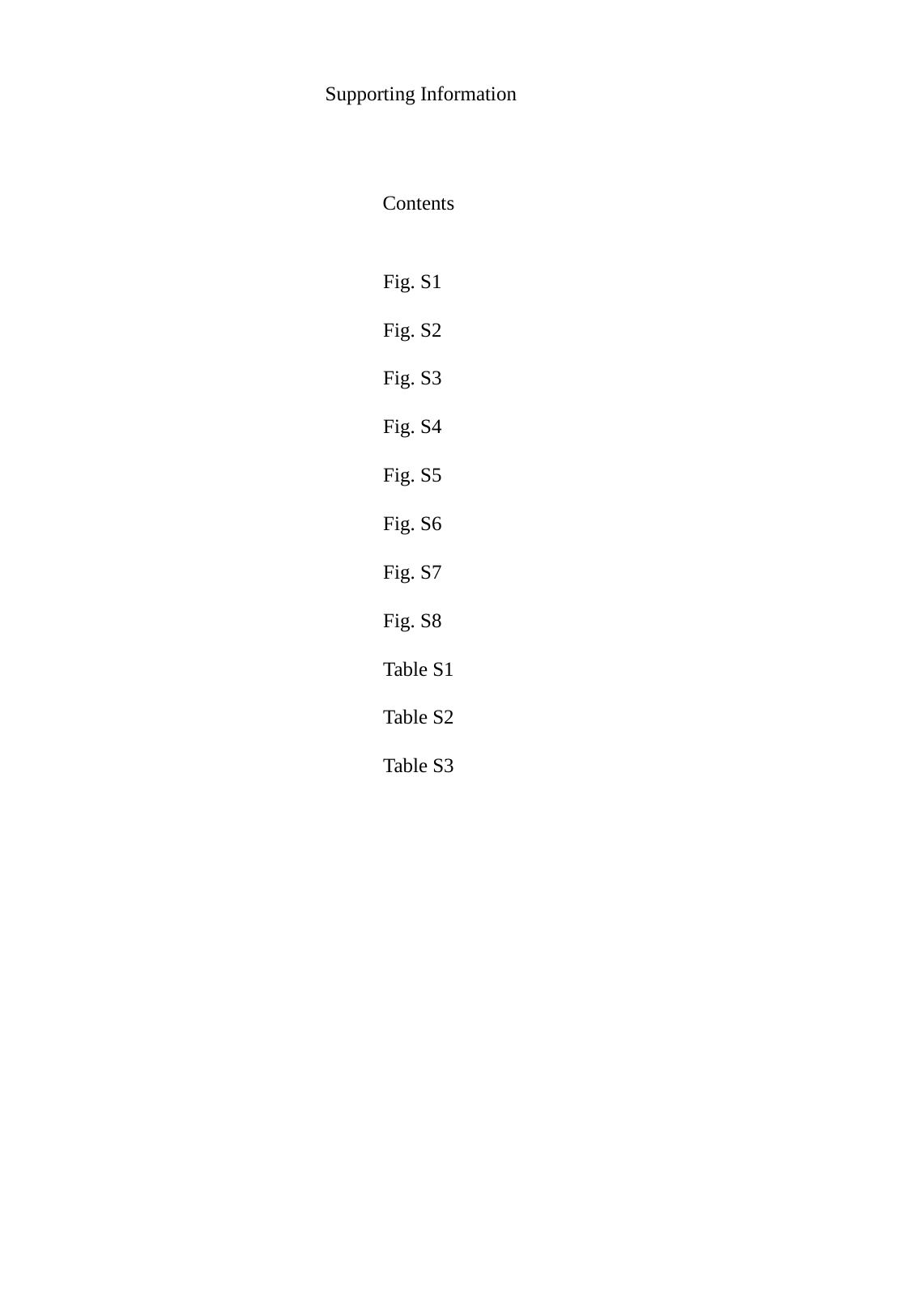

Supporting Information
Contents
Fig. S1
Fig. S2
Fig. S3
Fig. S4
Fig. S5
Fig. S6
Fig. S7
Fig. S8
Table S1
Table S2
Table S3

## Slide 2
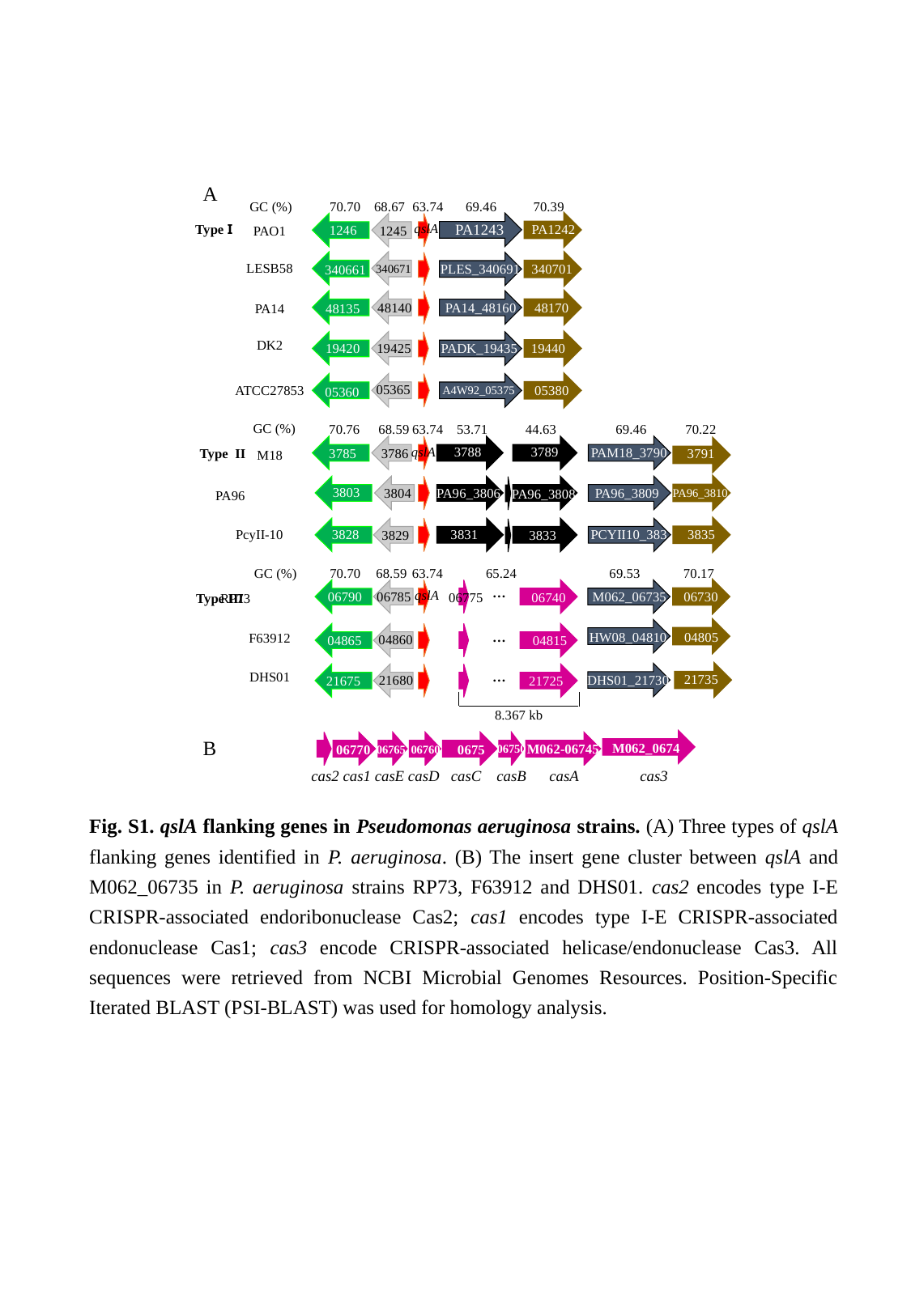

A
GC (%)
70.70
68.67
63.74
69.46
70.39
PA1243
qslA
PA1242
Type I
1246
PAO1
1245
LESB58
PLES_340691
340701
340671
340661
48140
PA14_48160
48170
48135
PA14
DK2
19425
PADK_19435
19440
19420
05365
05380
ATCC27853
A4W92_05375
05360
GC (%)
70.76
68.59
63.74
53.71
44.63
69.46
70.22
qslA
3788
3789
PAM18_3790
3785
3786
Type II
3791
M18
3803
3804
PA96_3806
PA96_3809
PA96_3810
PA96_3808
PA96
3835
3828
3831
PCYII10_3834
PcyII-10
3829
3833
GC (%)
70.70
68.59
63.74
65.24
69.53
70.17
…
qslA
06790
06785
M062_06735
06730
06775
06740
Type III
RP73
HW08_04810
…
04805
F63912
04860
04865
04815
…
DHS01
21735
DHS01_21730
21680
21675
21725
8.367 kb
B
M062_06740
M062-06745
06770
06755
06750
06765
06760
cas2 cas1 casE casD casC casB casA cas3
Fig. S1. qslA flanking genes in Pseudomonas aeruginosa strains. (A) Three types of qslA flanking genes identified in P. aeruginosa. (B) The insert gene cluster between qslA and M062_06735 in P. aeruginosa strains RP73, F63912 and DHS01. cas2 encodes type I-E CRISPR-associated endoribonuclease Cas2; cas1 encodes type I-E CRISPR-associated endonuclease Cas1; cas3 encode CRISPR-associated helicase/endonuclease Cas3. All sequences were retrieved from NCBI Microbial Genomes Resources. Position-Specific Iterated BLAST (PSI-BLAST) was used for homology analysis.

## Slide 3
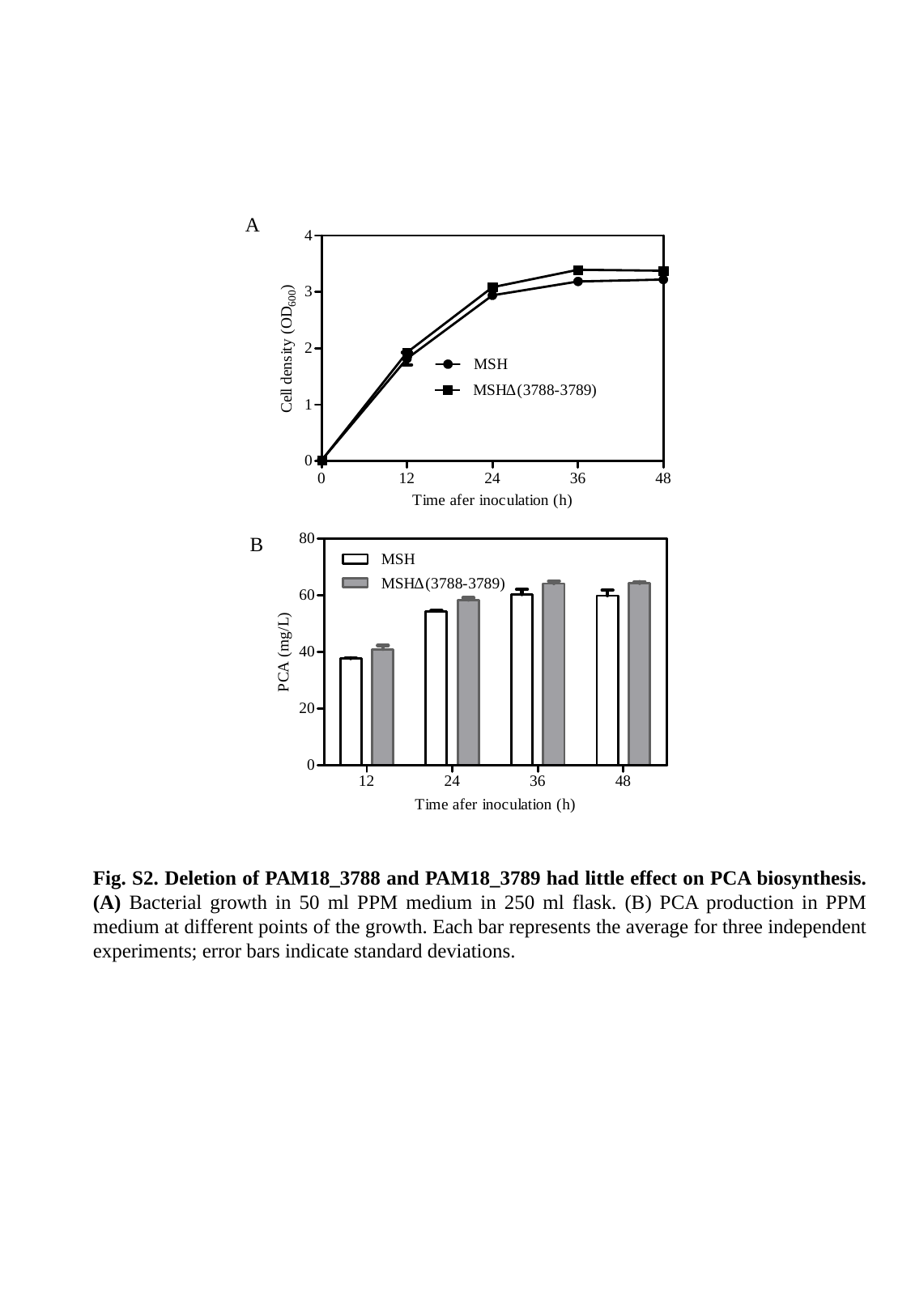

A
B
Fig. S2. Deletion of PAM18_3788 and PAM18_3789 had little effect on PCA biosynthesis. (A) Bacterial growth in 50 ml PPM medium in 250 ml flask. (B) PCA production in PPM medium at different points of the growth. Each bar represents the average for three independent experiments; error bars indicate standard deviations.

## Slide 4
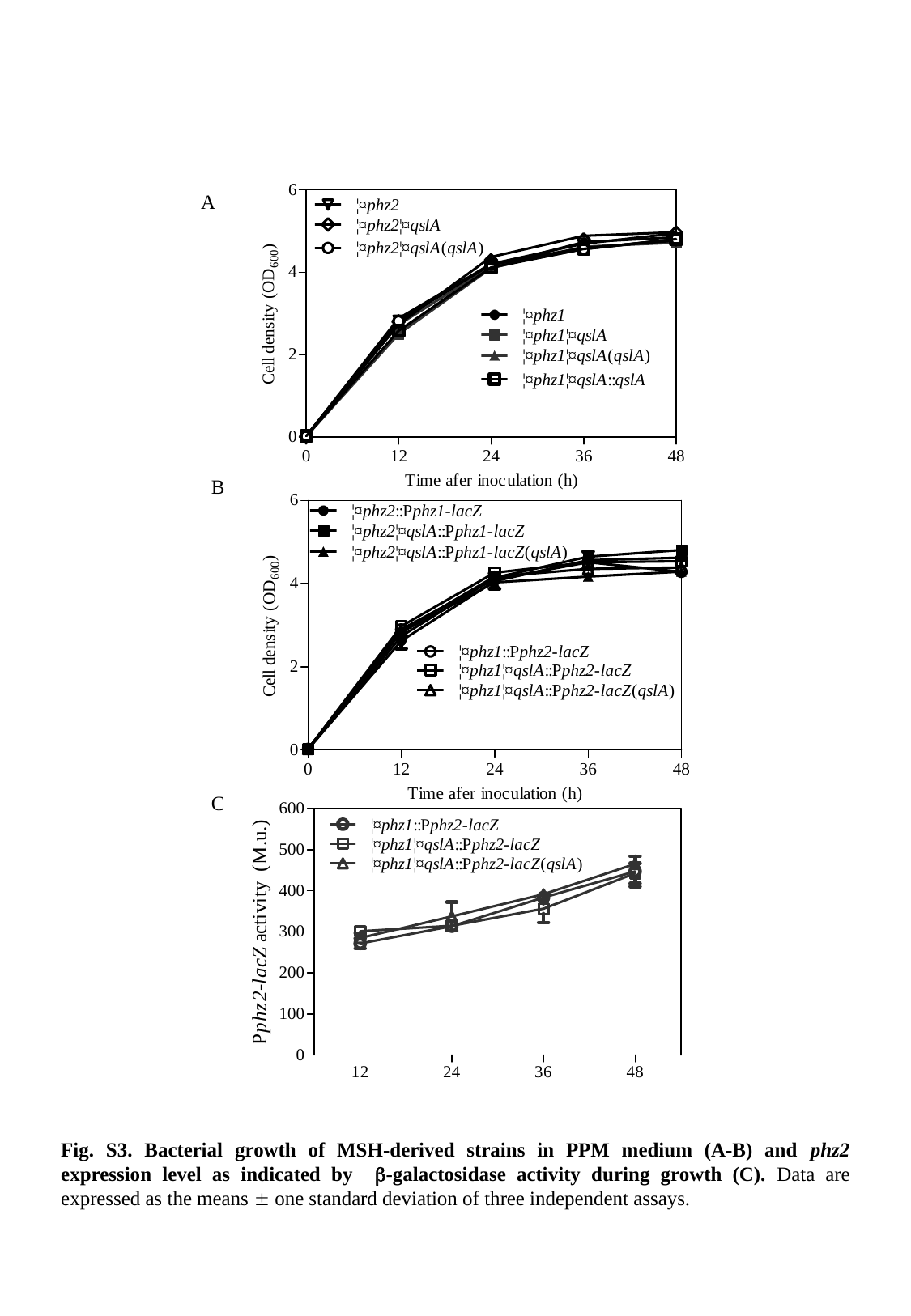

A
B
C
Fig. S3. Bacterial growth of MSH-derived strains in PPM medium (A-B) and phz2 expression level as indicated by β-galactosidase activity during growth (C). Data are expressed as the means ± one standard deviation of three independent assays.

## Slide 5
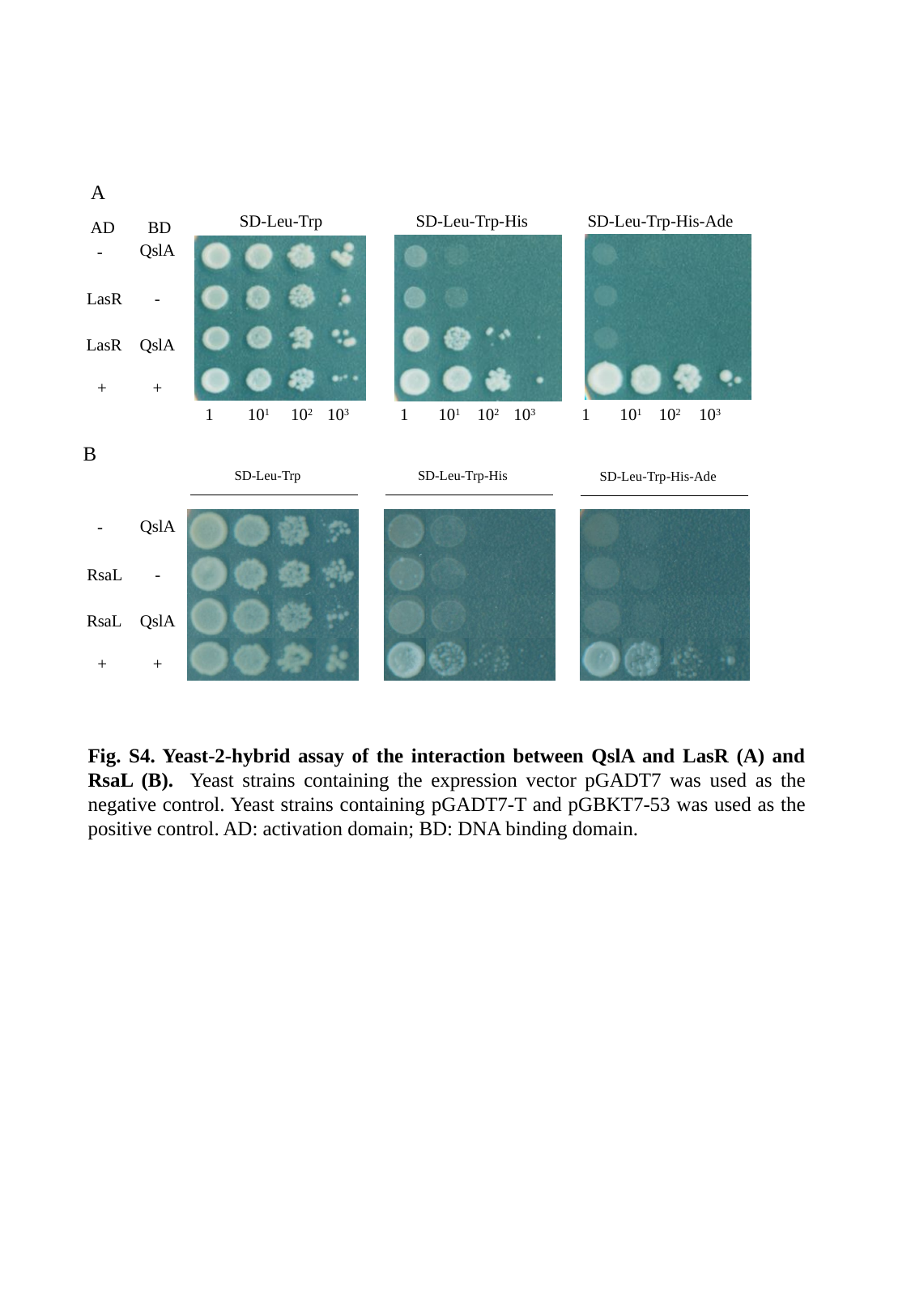

A
SD-Leu-Trp SD-Leu-Trp-His SD-Leu-Trp-His-Ade
AD
BD
QslA
-
LasR
-
LasR
QslA
+
+
1 101 102 103 1 101 102 103 1 101 102 103
B
SD-Leu-Trp
SD-Leu-Trp-His
SD-Leu-Trp-His-Ade
QslA
-
RsaL
-
RsaL
QslA
+
+
Fig. S4. Yeast-2-hybrid assay of the interaction between QslA and LasR (A) and RsaL (B). Yeast strains containing the expression vector pGADT7 was used as the negative control. Yeast strains containing pGADT7-T and pGBKT7-53 was used as the positive control. AD: activation domain; BD: DNA binding domain.

## Slide 6
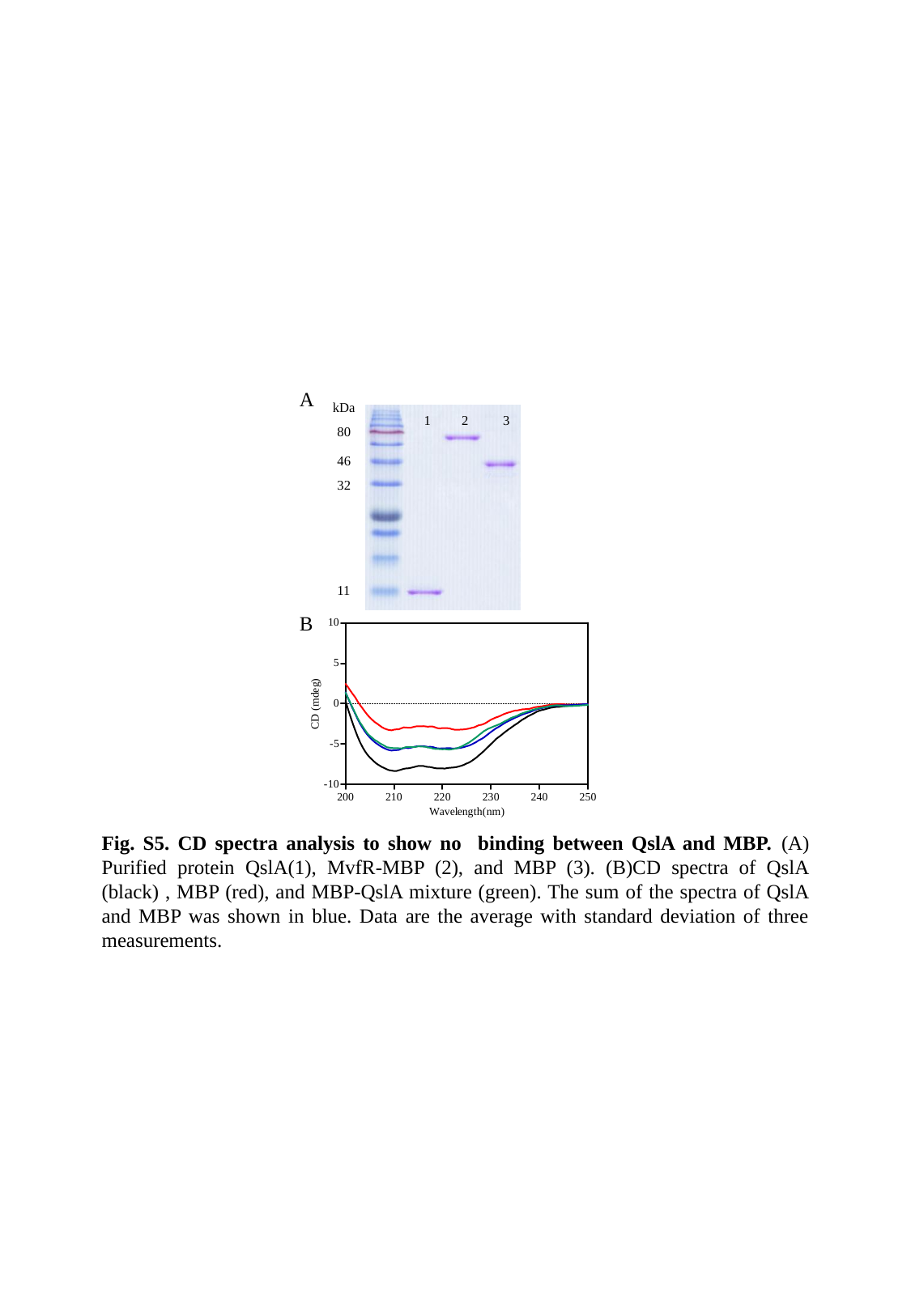

A
kDa
1 2 3
80
46
32
11
B
Fig. S5. CD spectra analysis to show no binding between QslA and MBP. (A) Purified protein QslA(1), MvfR-MBP (2), and MBP (3). (B)CD spectra of QslA (black) , MBP (red), and MBP-QslA mixture (green). The sum of the spectra of QslA and MBP was shown in blue. Data are the average with standard deviation of three measurements.

## Slide 7
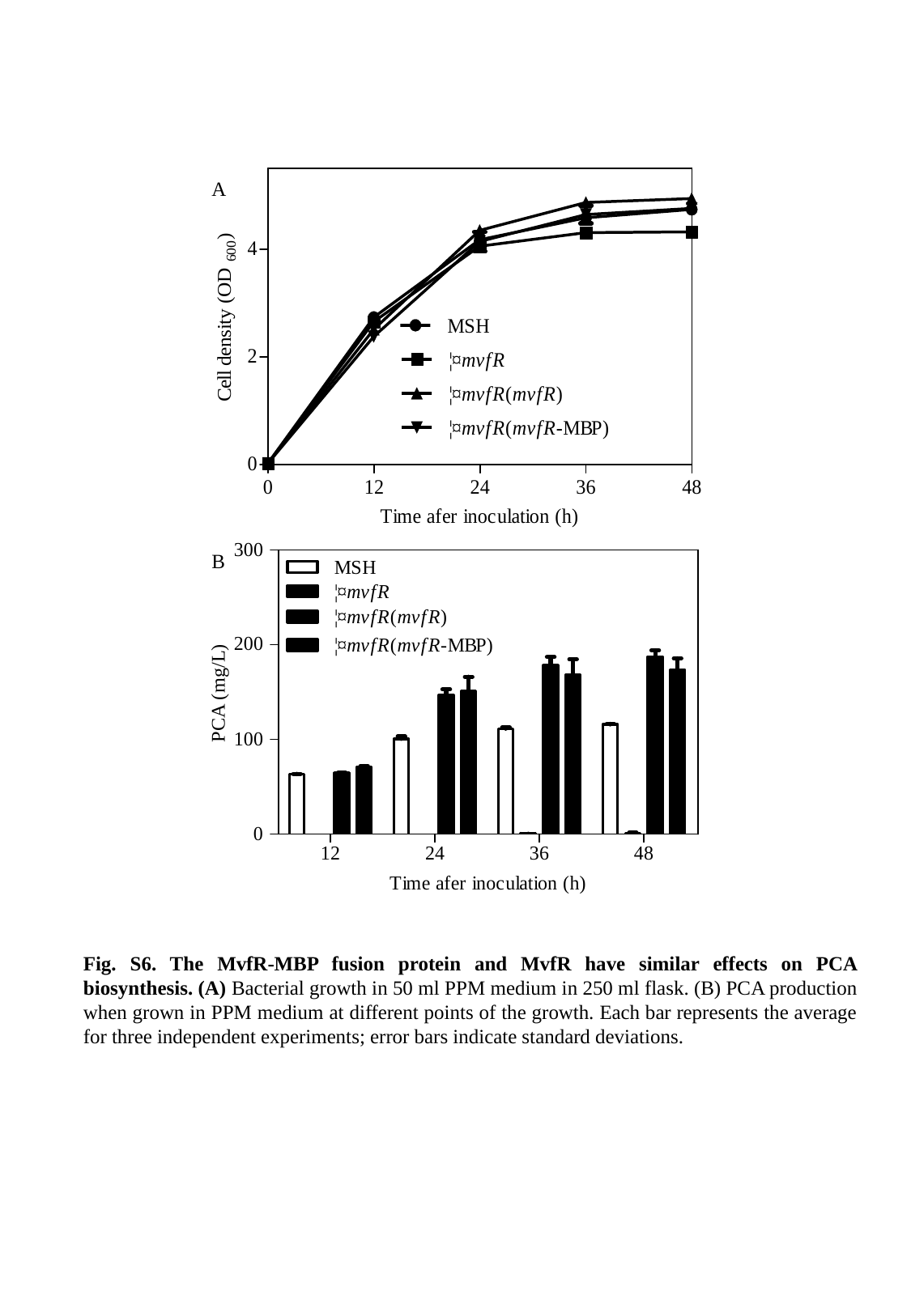

A
B
Fig. S6. The MvfR-MBP fusion protein and MvfR have similar effects on PCA biosynthesis. (A) Bacterial growth in 50 ml PPM medium in 250 ml flask. (B) PCA production when grown in PPM medium at different points of the growth. Each bar represents the average for three independent experiments; error bars indicate standard deviations.

## Slide 8
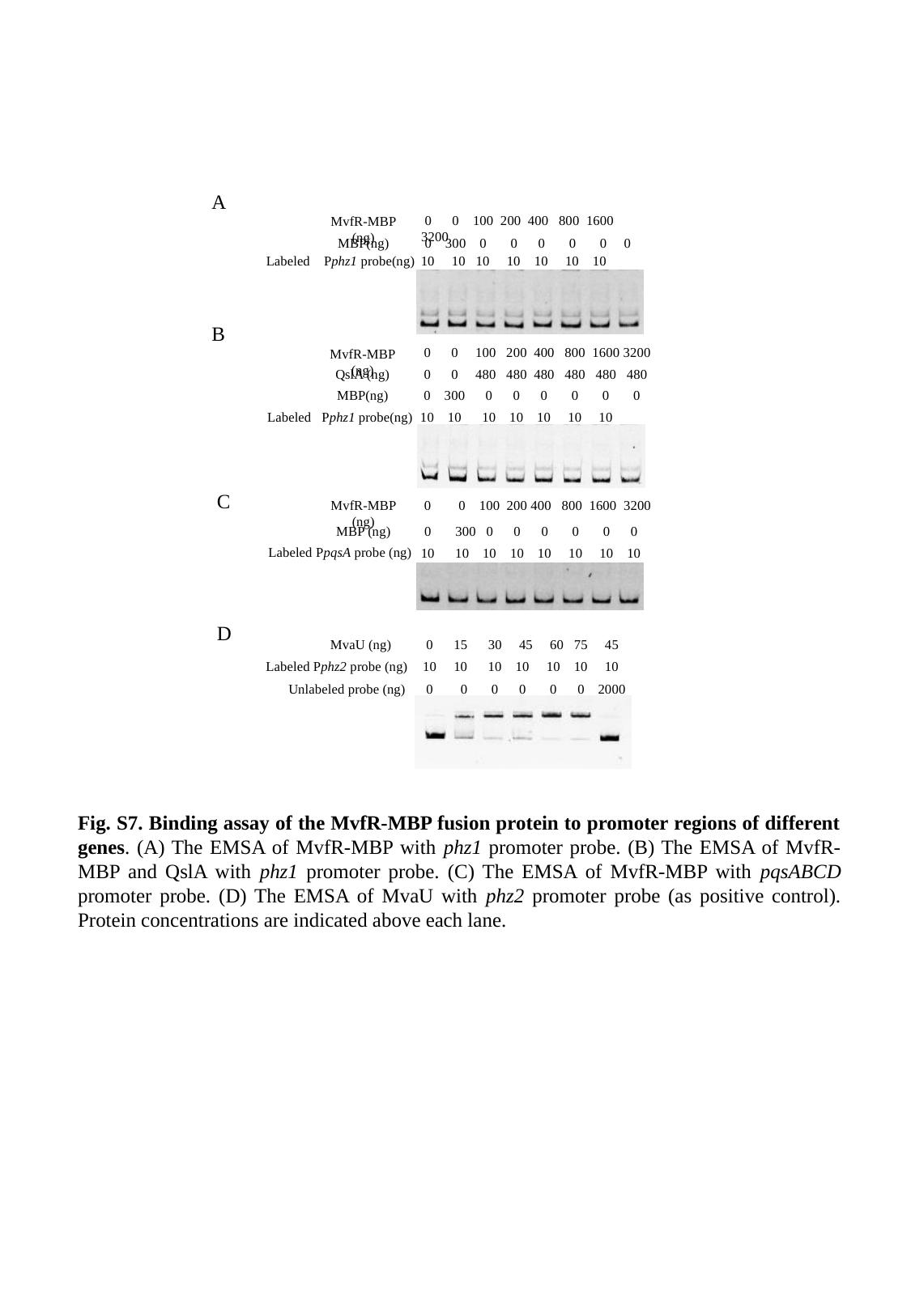

A
 0 0 100 200 400 800 1600 3200
MvfR-MBP (ng)
MBP(ng)
Labeled Pphz1 probe(ng)
 0 300 0 0 0 0 0 0
10 10 10 10 10 10 10 10
B
 0 0 100 200 400 800 1600 3200
MvfR-MBP (ng)
QslA (ng)
 0 0 480 480 480 480 480 480
MBP(ng)
 0 300 0 0 0 0 0 0
Labeled Pphz1 probe(ng)
10 10 10 10 10 10 10 10
C
MvfR-MBP (ng)
 0 0 100 200 400 800 1600 3200
MBP (ng)
 0 300 0 0 0 0 0 0
Labeled PpqsA probe (ng)
10 10 10 10 10 10 10 10
D
 0 15 30 45 60 75 45
MvaU (ng)
Labeled Pphz2 probe (ng)
 10 10 10 10 10 10 10
 0 0 0 0 0 0 2000
Unlabeled probe (ng)
Fig. S7. Binding assay of the MvfR-MBP fusion protein to promoter regions of different genes. (A) The EMSA of MvfR-MBP with phz1 promoter probe. (B) The EMSA of MvfR-MBP and QslA with phz1 promoter probe. (C) The EMSA of MvfR-MBP with pqsABCD promoter probe. (D) The EMSA of MvaU with phz2 promoter probe (as positive control). Protein concentrations are indicated above each lane.

## Slide 9
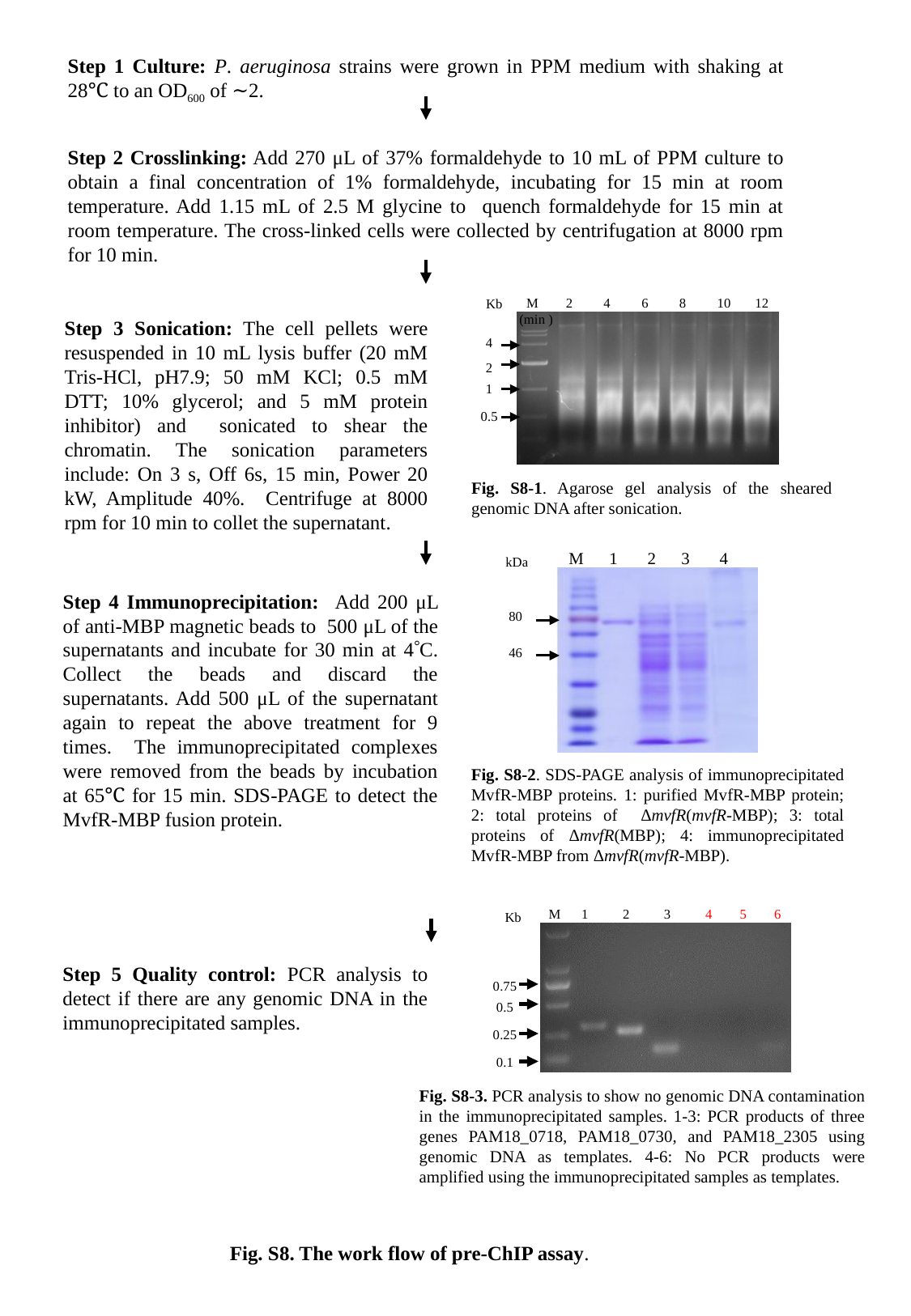

Step 1 Culture: P. aeruginosa strains were grown in PPM medium with shaking at 28℃ to an OD600 of ∼2.
Step 2 Crosslinking: Add 270 μL of 37% formaldehyde to 10 mL of PPM culture to obtain a final concentration of 1% formaldehyde, incubating for 15 min at room temperature. Add 1.15 mL of 2.5 M glycine to quench formaldehyde for 15 min at room temperature. The cross-linked cells were collected by centrifugation at 8000 rpm for 10 min.
 M 2 4 6 8 10 12 (min )
Kb
4
2
1
0.5
Step 3 Sonication: The cell pellets were resuspended in 10 mL lysis buffer (20 mM Tris-HCl, pH7.9; 50 mM KCl; 0.5 mM DTT; 10% glycerol; and 5 mM protein inhibitor) and sonicated to shear the chromatin. The sonication parameters include: On 3 s, Off 6s, 15 min, Power 20 kW, Amplitude 40%. Centrifuge at 8000 rpm for 10 min to collet the supernatant.
Fig. S8-1. Agarose gel analysis of the sheared genomic DNA after sonication.
M 1 2 3 4
kDa
80
46
Step 4 Immunoprecipitation: Add 200 μL of anti-MBP magnetic beads to 500 μL of the supernatants and incubate for 30 min at 4°C. Collect the beads and discard the supernatants. Add 500 μL of the supernatant again to repeat the above treatment for 9 times. The immunoprecipitated complexes were removed from the beads by incubation at 65℃ for 15 min. SDS-PAGE to detect the MvfR-MBP fusion protein.
Fig. S8-2. SDS-PAGE analysis of immunoprecipitated MvfR-MBP proteins. 1: purified MvfR-MBP protein; 2: total proteins of ΔmvfR(mvfR-MBP); 3: total proteins of ΔmvfR(MBP); 4: immunoprecipitated MvfR-MBP from ΔmvfR(mvfR-MBP).
M 1 2 3 4 5 6
Kb
0.75
0.5
0.25
0.1
Step 5 Quality control: PCR analysis to detect if there are any genomic DNA in the immunoprecipitated samples.
Fig. S8-3. PCR analysis to show no genomic DNA contamination in the immunoprecipitated samples. 1-3: PCR products of three genes PAM18_0718, PAM18_0730, and PAM18_2305 using genomic DNA as templates. 4-6: No PCR products were amplified using the immunoprecipitated samples as templates.
Fig. S8. The work flow of pre-ChIP assay.

## Slide 10
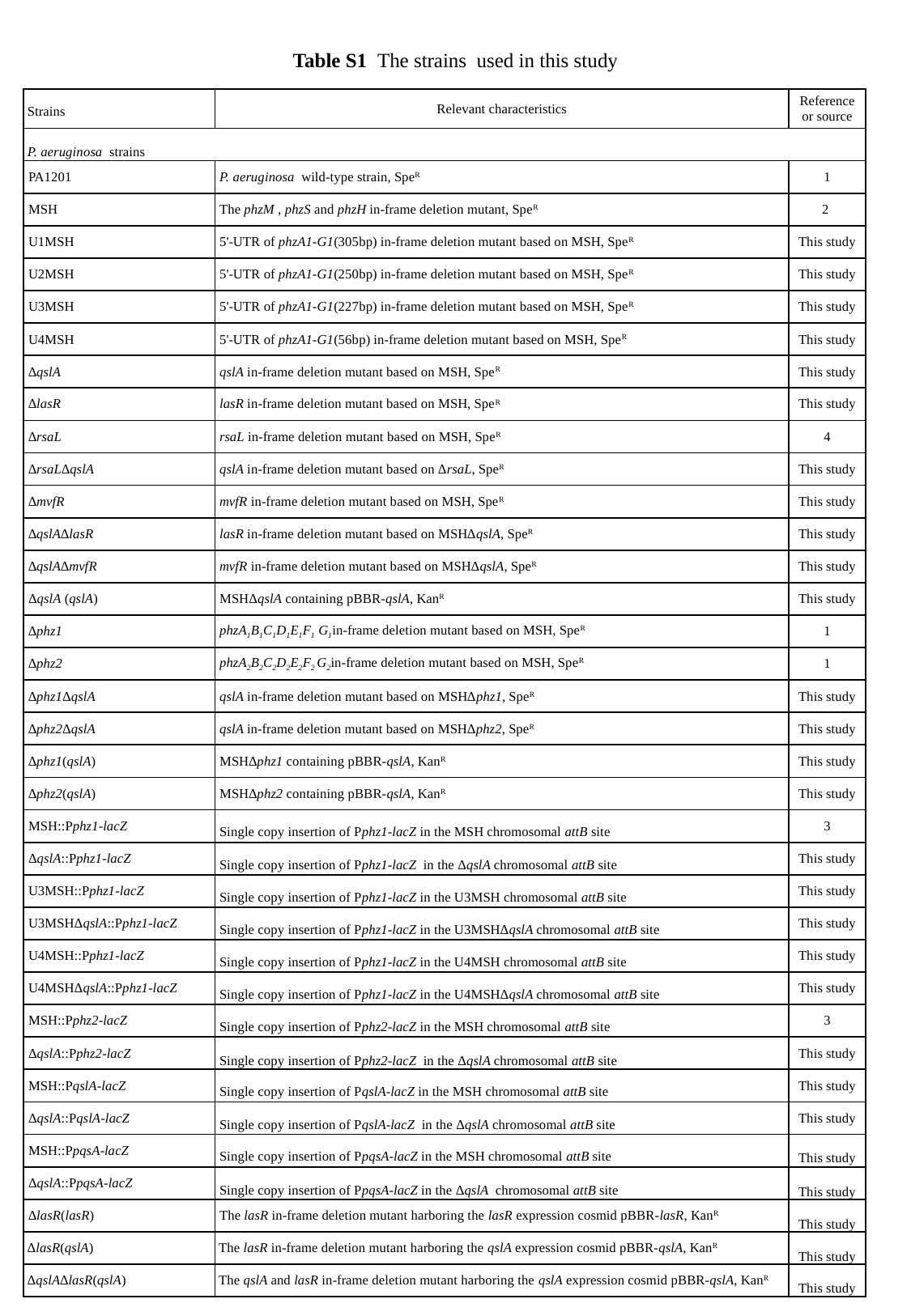

Table S1 The strains used in this study
| Strains | Relevant characteristics | Reference or source |
| --- | --- | --- |
| P. aeruginosa strains | | |
| PA1201 | P. aeruginosa wild-type strain, SpeR | 1 |
| MSH | The phzM , phzS and phzH in-frame deletion mutant, SpeR | 2 |
| U1MSH | 5'-UTR of phzA1-G1(305bp) in-frame deletion mutant based on MSH, SpeR | This study |
| U2MSH | 5'-UTR of phzA1-G1(250bp) in-frame deletion mutant based on MSH, SpeR | This study |
| U3MSH | 5'-UTR of phzA1-G1(227bp) in-frame deletion mutant based on MSH, SpeR | This study |
| U4MSH | 5'-UTR of phzA1-G1(56bp) in-frame deletion mutant based on MSH, SpeR | This study |
| ∆qslA | qslA in-frame deletion mutant based on MSH, SpeR | This study |
| ∆lasR | lasR in-frame deletion mutant based on MSH, SpeR | This study |
| ∆rsaL | rsaL in-frame deletion mutant based on MSH, SpeR | 4 |
| ∆rsaL∆qslA | qslA in-frame deletion mutant based on ∆rsaL, SpeR | This study |
| ∆mvfR | mvfR in-frame deletion mutant based on MSH, SpeR | This study |
| ∆qslA∆lasR | lasR in-frame deletion mutant based on MSH∆qslA, SpeR | This study |
| ∆qslA∆mvfR | mvfR in-frame deletion mutant based on MSH∆qslA, SpeR | This study |
| ΔqslA (qslA) | MSHΔqslA containing pBBR-qslA, KanR | This study |
| ∆phz1 | phzA1B1C1D1E1F1 G1in-frame deletion mutant based on MSH, SpeR | 1 |
| ∆phz2 | phzA2B2C2D2E2F2 G2in-frame deletion mutant based on MSH, SpeR | 1 |
| ∆phz1ΔqslA | qslA in-frame deletion mutant based on MSH∆phz1, SpeR | This study |
| ∆phz2ΔqslA | qslA in-frame deletion mutant based on MSH∆phz2, SpeR | This study |
| ∆phz1(qslA) | MSH∆phz1 containing pBBR-qslA, KanR | This study |
| ∆phz2(qslA) | MSH∆phz2 containing pBBR-qslA, KanR | This study |
| MSH::Pphz1-lacZ | Single copy insertion of Pphz1-lacZ in the MSH chromosomal attB site | 3 |
| ΔqslA::Pphz1-lacZ | Single copy insertion of Pphz1-lacZ in the ΔqslA chromosomal attB site | This study |
| U3MSH::Pphz1-lacZ | Single copy insertion of Pphz1-lacZ in the U3MSH chromosomal attB site | This study |
| U3MSHΔqslA::Pphz1-lacZ | Single copy insertion of Pphz1-lacZ in the U3MSHΔqslA chromosomal attB site | This study |
| U4MSH::Pphz1-lacZ | Single copy insertion of Pphz1-lacZ in the U4MSH chromosomal attB site | This study |
| U4MSHΔqslA::Pphz1-lacZ | Single copy insertion of Pphz1-lacZ in the U4MSHΔqslA chromosomal attB site | This study |
| MSH::Pphz2-lacZ | Single copy insertion of Pphz2-lacZ in the MSH chromosomal attB site | 3 |
| ΔqslA::Pphz2-lacZ | Single copy insertion of Pphz2-lacZ in the ΔqslA chromosomal attB site | This study |
| MSH::PqslA-lacZ | Single copy insertion of PqslA-lacZ in the MSH chromosomal attB site | This study |
| ΔqslA::PqslA-lacZ | Single copy insertion of PqslA-lacZ in the ΔqslA chromosomal attB site | This study |
| MSH::PpqsA-lacZ | Single copy insertion of PpqsA-lacZ in the MSH chromosomal attB site | This study |
| ΔqslA::PpqsA-lacZ | Single copy insertion of PpqsA-lacZ in the ΔqslA chromosomal attB site | This study |
| ∆lasR(lasR) | The lasR in-frame deletion mutant harboring the lasR expression cosmid pBBR-lasR, KanR | This study |
| ∆lasR(qslA) | The lasR in-frame deletion mutant harboring the qslA expression cosmid pBBR-qslA, KanR | This study |
| ∆qslA∆lasR(qslA) | The qslA and lasR in-frame deletion mutant harboring the qslA expression cosmid pBBR-qslA, KanR | This study |

## Slide 11
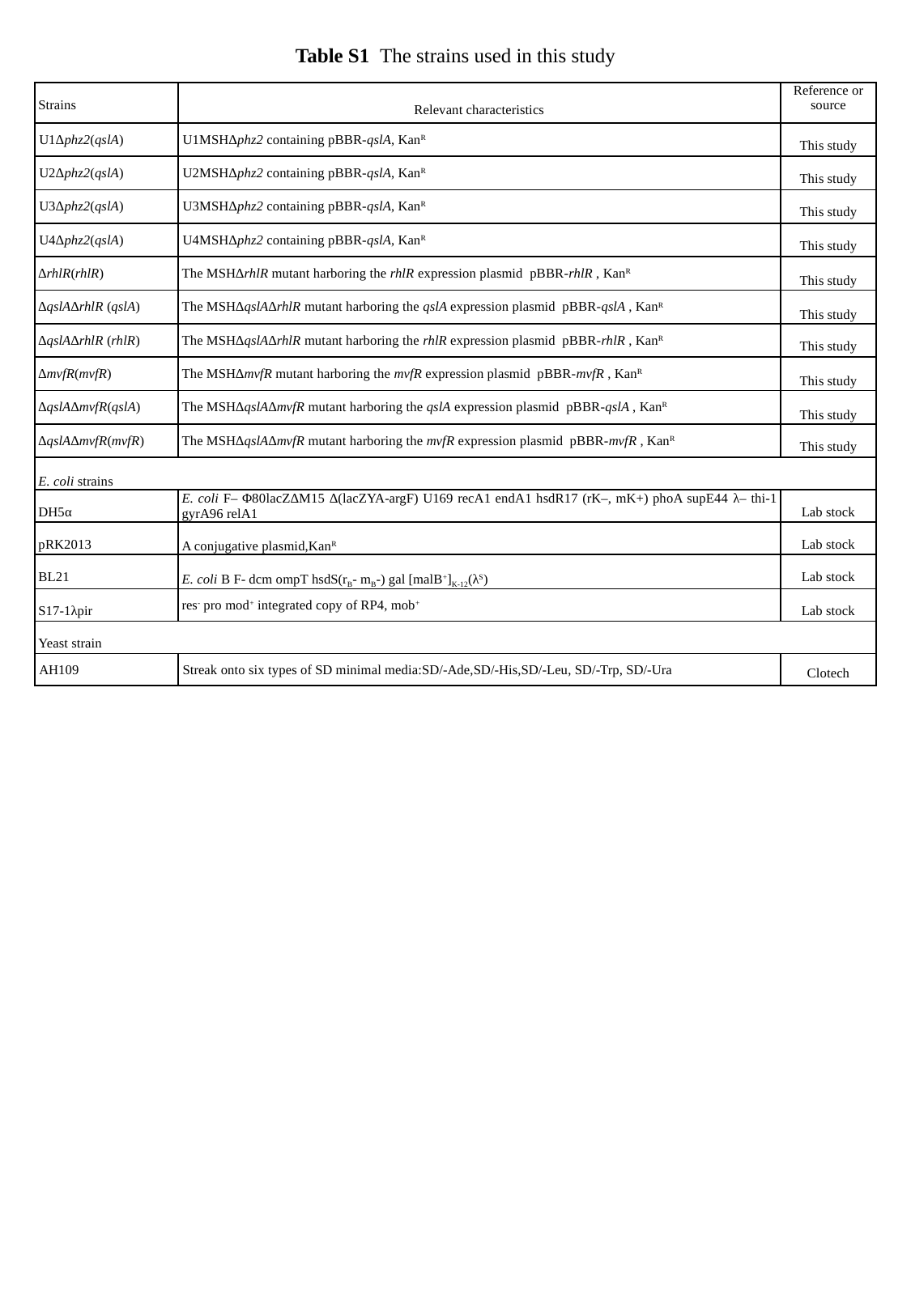

Table S1 The strains used in this study
| Strains | Relevant characteristics | Reference or source |
| --- | --- | --- |
| U1Δphz2(qslA) | U1MSHΔphz2 containing pBBR-qslA, KanR | This study |
| U2Δphz2(qslA) | U2MSHΔphz2 containing pBBR-qslA, KanR | This study |
| U3Δphz2(qslA) | U3MSHΔphz2 containing pBBR-qslA, KanR | This study |
| U4Δphz2(qslA) | U4MSHΔphz2 containing pBBR-qslA, KanR | This study |
| ∆rhlR(rhlR) | The MSH∆rhlR mutant harboring the rhlR expression plasmid pBBR-rhlR , KanR | This study |
| ∆qslA∆rhlR (qslA) | The MSH∆qslA∆rhlR mutant harboring the qslA expression plasmid pBBR-qslA , KanR | This study |
| ∆qslA∆rhlR (rhlR) | The MSH∆qslA∆rhlR mutant harboring the rhlR expression plasmid pBBR-rhlR , KanR | This study |
| ∆mvfR(mvfR) | The MSH∆mvfR mutant harboring the mvfR expression plasmid pBBR-mvfR , KanR | This study |
| ∆qslA∆mvfR(qslA) | The MSH∆qslA∆mvfR mutant harboring the qslA expression plasmid pBBR-qslA , KanR | This study |
| ∆qslA∆mvfR(mvfR) | The MSH∆qslA∆mvfR mutant harboring the mvfR expression plasmid pBBR-mvfR , KanR | This study |
| E. coli strains | | |
| DH5α | E. coli F– Φ80lacZΔM15 Δ(lacZYA-argF) U169 recA1 endA1 hsdR17 (rK–, mK+) phoA supE44 λ– thi-1 gyrA96 relA1 | Lab stock |
| pRK2013 | A conjugative plasmid,KanR | Lab stock |
| BL21 | E. coli B F- dcm ompT hsdS(rB- mB-) gal [malB+]K-12(λS) | Lab stock |
| S17-1λpir | res- pro mod+ integrated copy of RP4, mob+ | Lab stock |
| Yeast strain | | |
| AH109 | Streak onto six types of SD minimal media:SD/-Ade,SD/-His,SD/-Leu, SD/-Trp, SD/-Ura | Clotech |

## Slide 12
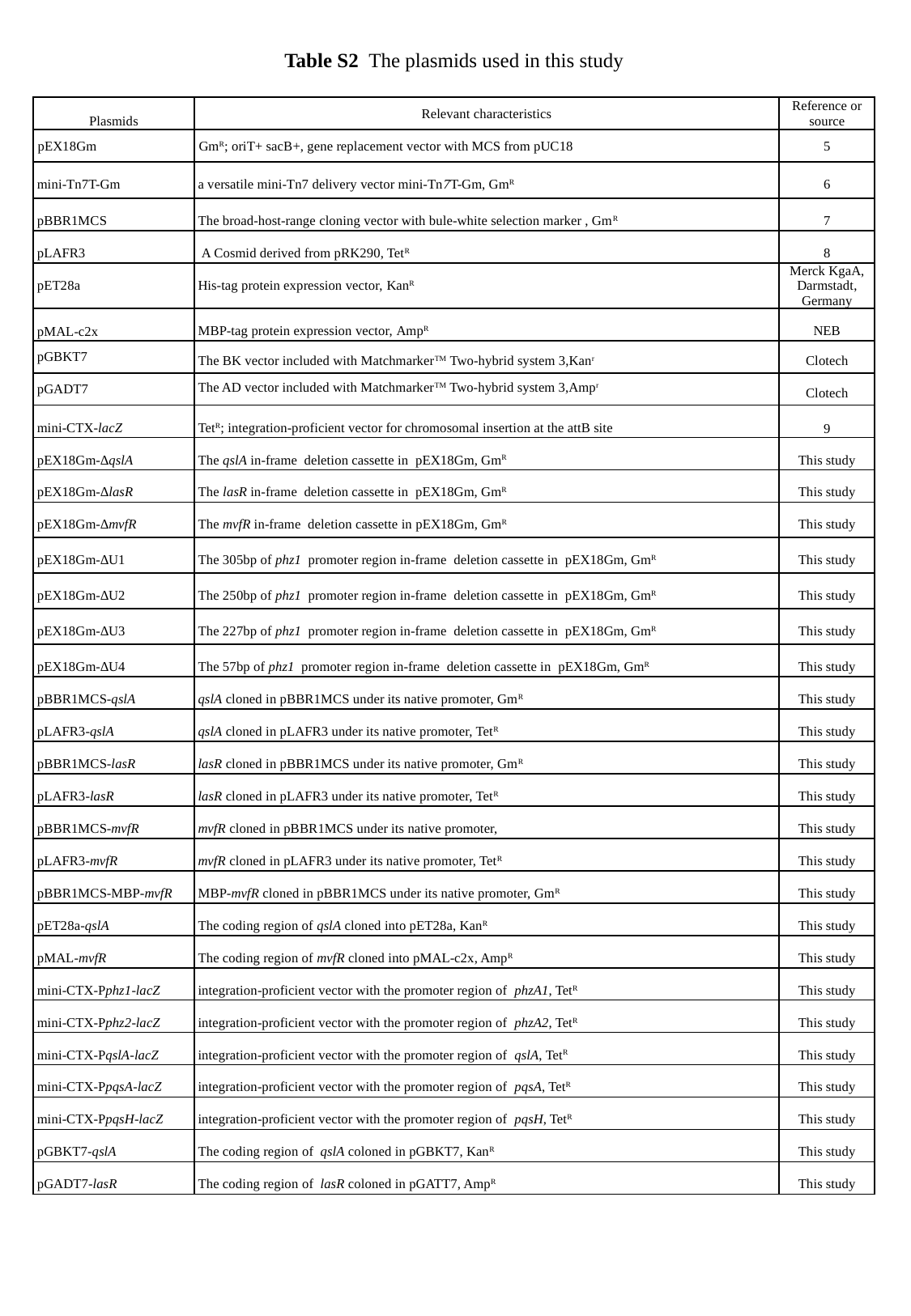

Table S2 The plasmids used in this study
| Plasmids | Relevant characteristics | Reference or source |
| --- | --- | --- |
| pEX18Gm | GmR; oriT+ sacB+, gene replacement vector with MCS from pUC18 | 5 |
| mini-Tn7T-Gm | a versatile mini-Tn7 delivery vector mini-Tn7T-Gm, GmR | 6 |
| pBBR1MCS | The broad-host-range cloning vector with bule-white selection marker , GmR | 7 |
| pLAFR3 | A Cosmid derived from pRK290, TetR | 8 |
| pET28a | His-tag protein expression vector, KanR | Merck KgaA, Darmstadt, Germany |
| pMAL-c2x | MBP-tag protein expression vector, AmpR | NEB |
| pGBKT7 | The BK vector included with MatchmarkerTM Two-hybrid system 3,Kanr | Clotech |
| pGADT7 | The AD vector included with MatchmarkerTM Two-hybrid system 3,Ampr | Clotech |
| mini-CTX-lacZ | TetR; integration-proficient vector for chromosomal insertion at the attB site | 9 |
| pEX18Gm-∆qslA | The qslA in-frame deletion cassette in pEX18Gm, GmR | This study |
| pEX18Gm-∆lasR | The lasR in-frame deletion cassette in pEX18Gm, GmR | This study |
| pEX18Gm-∆mvfR | The mvfR in-frame deletion cassette in pEX18Gm, GmR | This study |
| pEX18Gm-∆U1 | The 305bp of phz1 promoter region in-frame deletion cassette in pEX18Gm, GmR | This study |
| pEX18Gm-∆U2 | The 250bp of phz1 promoter region in-frame deletion cassette in pEX18Gm, GmR | This study |
| pEX18Gm-∆U3 | The 227bp of phz1 promoter region in-frame deletion cassette in pEX18Gm, GmR | This study |
| pEX18Gm-∆U4 | The 57bp of phz1 promoter region in-frame deletion cassette in pEX18Gm, GmR | This study |
| pBBR1MCS-qslA | qslA cloned in pBBR1MCS under its native promoter, GmR | This study |
| pLAFR3-qslA | qslA cloned in pLAFR3 under its native promoter, TetR | This study |
| pBBR1MCS-lasR | lasR cloned in pBBR1MCS under its native promoter, GmR | This study |
| pLAFR3-lasR | lasR cloned in pLAFR3 under its native promoter, TetR | This study |
| pBBR1MCS-mvfR | mvfR cloned in pBBR1MCS under its native promoter, | This study |
| pLAFR3-mvfR | mvfR cloned in pLAFR3 under its native promoter, TetR | This study |
| pBBR1MCS-MBP-mvfR | MBP-mvfR cloned in pBBR1MCS under its native promoter, GmR | This study |
| pET28a-qslA | The coding region of qslA cloned into pET28a, KanR | This study |
| pMAL-mvfR | The coding region of mvfR cloned into pMAL-c2x, AmpR | This study |
| mini-CTX-Pphz1-lacZ | integration-proficient vector with the promoter region of phzA1, TetR | This study |
| mini-CTX-Pphz2-lacZ | integration-proficient vector with the promoter region of phzA2, TetR | This study |
| mini-CTX-PqslA-lacZ | integration-proficient vector with the promoter region of qslA, TetR | This study |
| mini-CTX-PpqsA-lacZ | integration-proficient vector with the promoter region of pqsA, TetR | This study |
| mini-CTX-PpqsH-lacZ | integration-proficient vector with the promoter region of pqsH, TetR | This study |
| pGBKT7-qslA | The coding region of qslA coloned in pGBKT7, KanR | This study |
| pGADT7-lasR | The coding region of lasR coloned in pGATT7, AmpR | This study |

## Slide 13
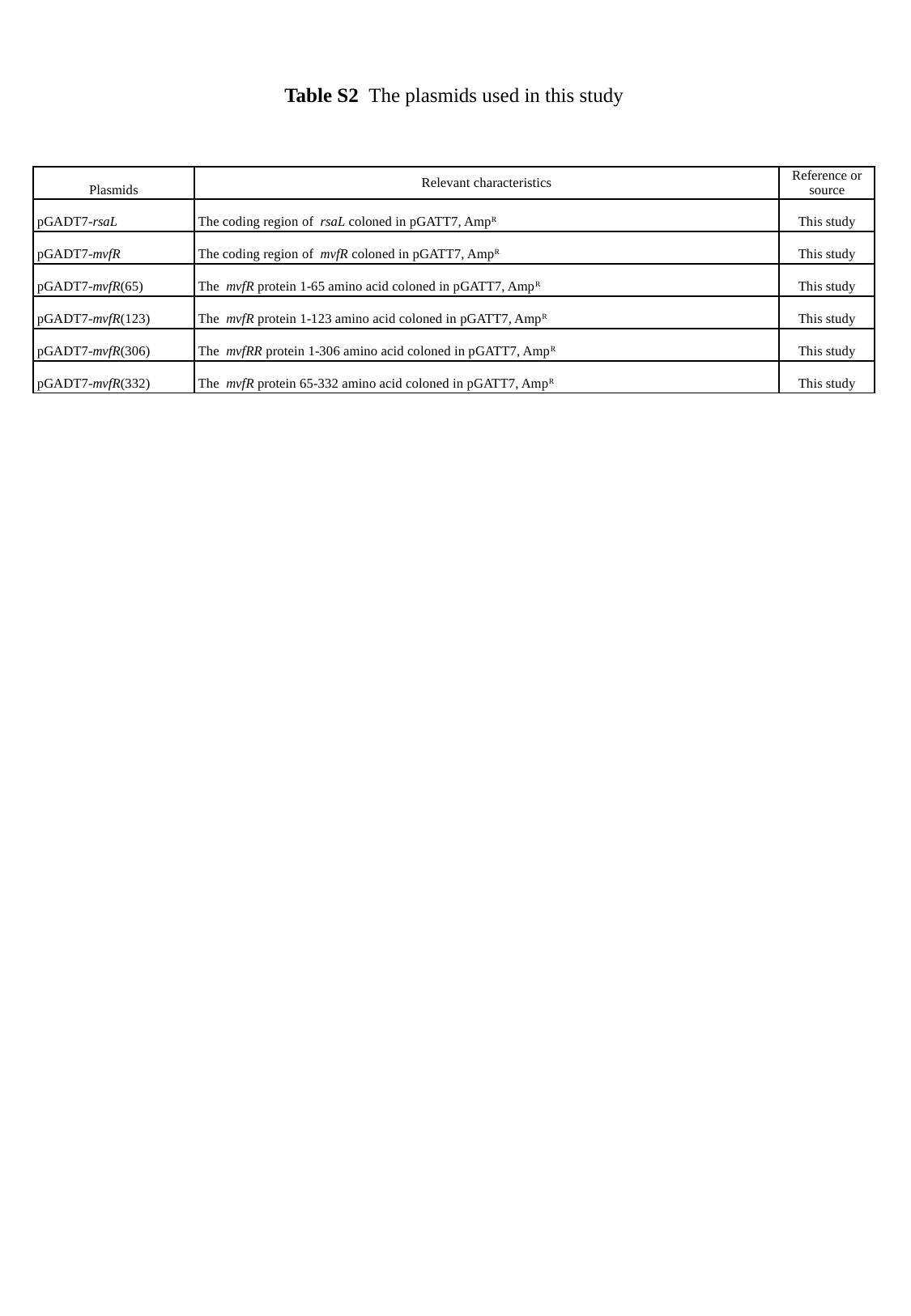

Table S2 The plasmids used in this study
| Plasmids | Relevant characteristics | Reference or source |
| --- | --- | --- |
| pGADT7-rsaL | The coding region of rsaL coloned in pGATT7, AmpR | This study |
| pGADT7-mvfR | The coding region of mvfR coloned in pGATT7, AmpR | This study |
| pGADT7-mvfR(65) | The mvfR protein 1-65 amino acid coloned in pGATT7, AmpR | This study |
| pGADT7-mvfR(123) | The mvfR protein 1-123 amino acid coloned in pGATT7, AmpR | This study |
| pGADT7-mvfR(306) | The mvfRR protein 1-306 amino acid coloned in pGATT7, AmpR | This study |
| pGADT7-mvfR(332) | The mvfR protein 65-332 amino acid coloned in pGATT7, AmpR | This study |

## Slide 14
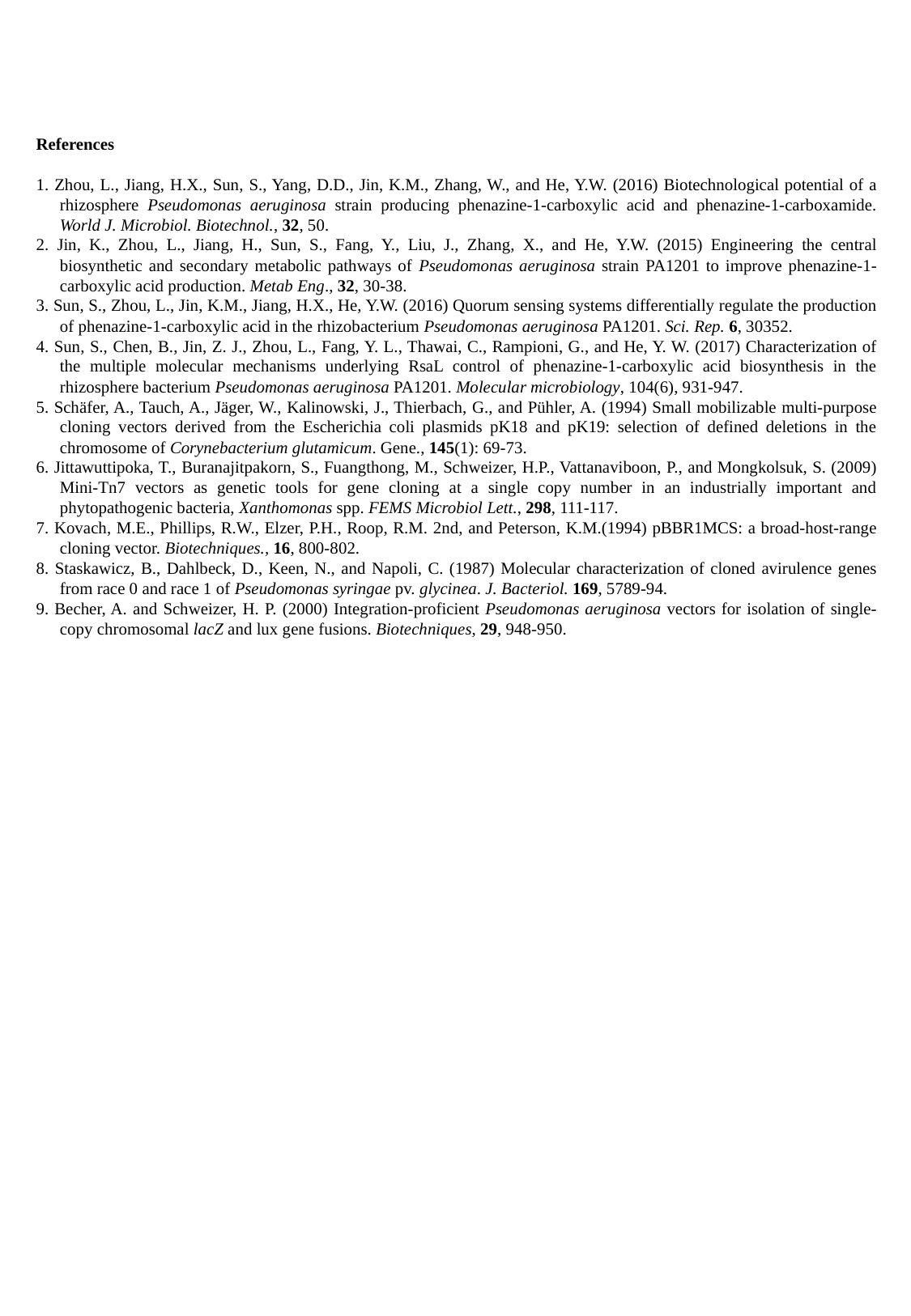

References
1. Zhou, L., Jiang, H.X., Sun, S., Yang, D.D., Jin, K.M., Zhang, W., and He, Y.W. (2016) Biotechnological potential of a rhizosphere Pseudomonas aeruginosa strain producing phenazine-1-carboxylic acid and phenazine-1-carboxamide. World J. Microbiol. Biotechnol., 32, 50.
2. Jin, K., Zhou, L., Jiang, H., Sun, S., Fang, Y., Liu, J., Zhang, X., and He, Y.W. (2015) Engineering the central biosynthetic and secondary metabolic pathways of Pseudomonas aeruginosa strain PA1201 to improve phenazine-1-carboxylic acid production. Metab Eng., 32, 30-38.
3. Sun, S., Zhou, L., Jin, K.M., Jiang, H.X., He, Y.W. (2016) Quorum sensing systems differentially regulate the production of phenazine-1-carboxylic acid in the rhizobacterium Pseudomonas aeruginosa PA1201. Sci. Rep. 6, 30352.
4. Sun, S., Chen, B., Jin, Z. J., Zhou, L., Fang, Y. L., Thawai, C., Rampioni, G., and He, Y. W. (2017) Characterization of the multiple molecular mechanisms underlying RsaL control of phenazine-1-carboxylic acid biosynthesis in the rhizosphere bacterium Pseudomonas aeruginosa PA1201. Molecular microbiology, 104(6), 931-947.
5. Schäfer, A., Tauch, A., Jäger, W., Kalinowski, J., Thierbach, G., and Pühler, A. (1994) Small mobilizable multi-purpose cloning vectors derived from the Escherichia coli plasmids pK18 and pK19: selection of defined deletions in the chromosome of Corynebacterium glutamicum. Gene., 145(1): 69-73.
6. Jittawuttipoka, T., Buranajitpakorn, S., Fuangthong, M., Schweizer, H.P., Vattanaviboon, P., and Mongkolsuk, S. (2009) Mini-Tn7 vectors as genetic tools for gene cloning at a single copy number in an industrially important and phytopathogenic bacteria, Xanthomonas spp. FEMS Microbiol Lett., 298, 111-117.
7. Kovach, M.E., Phillips, R.W., Elzer, P.H., Roop, R.M. 2nd, and Peterson, K.M.(1994) pBBR1MCS: a broad-host-range cloning vector. Biotechniques., 16, 800-802.
8. Staskawicz, B., Dahlbeck, D., Keen, N., and Napoli, C. (1987) Molecular characterization of cloned avirulence genes from race 0 and race 1 of Pseudomonas syringae pv. glycinea. J. Bacteriol. 169, 5789-94.
9. Becher, A. and Schweizer, H. P. (2000) Integration-proficient Pseudomonas aeruginosa vectors for isolation of single-copy chromosomal lacZ and lux gene fusions. Biotechniques, 29, 948-950.

## Slide 15
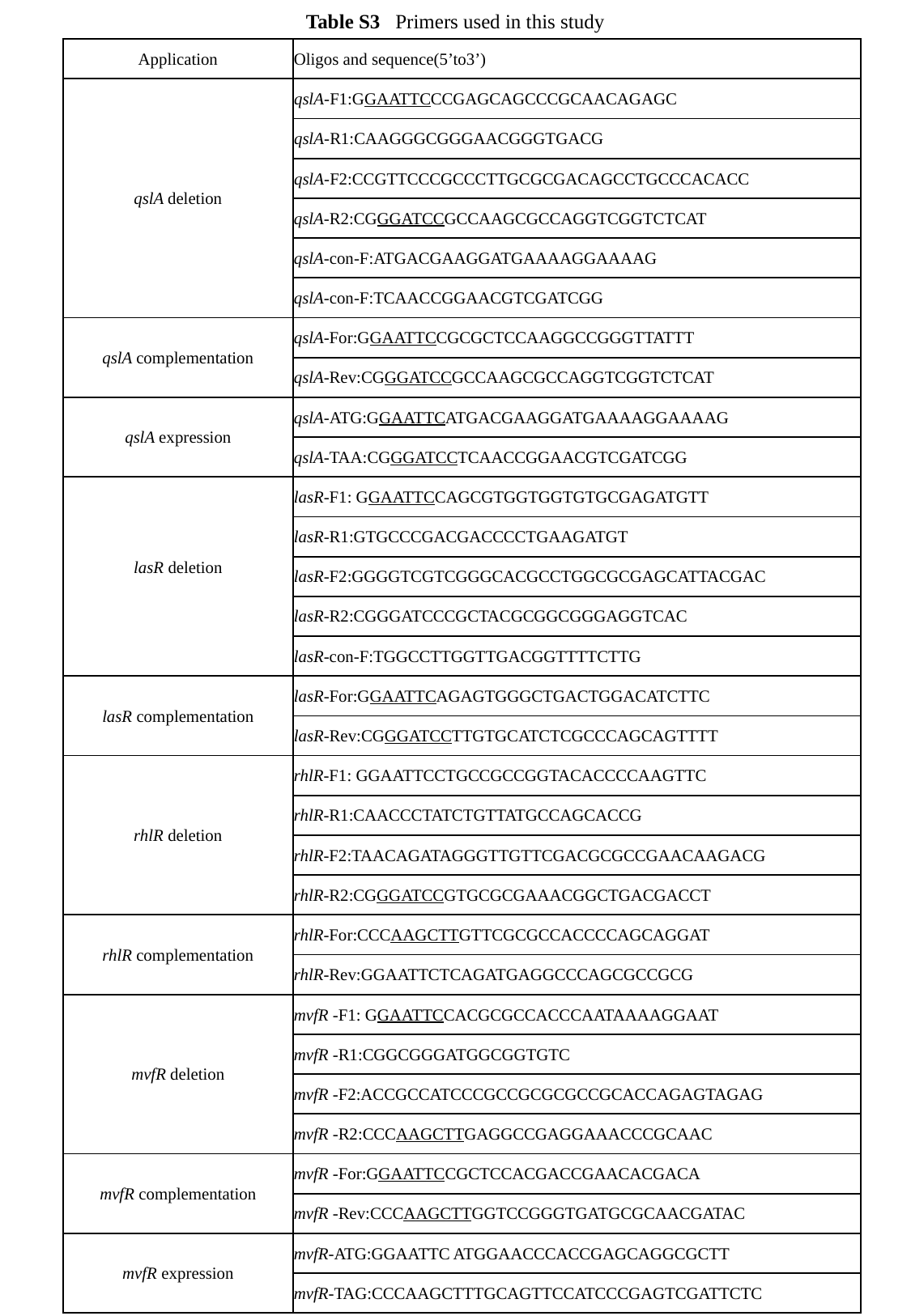

Table S3 Primers used in this study
| Application | Oligos and sequence(5’to3’) |
| --- | --- |
| qslA deletion | qslA-F1:GGAATTCCCGAGCAGCCCGCAACAGAGC |
| | qslA-R1:CAAGGGCGGGAACGGGTGACG |
| | qslA-F2:CCGTTCCCGCCCTTGCGCGACAGCCTGCCCACACC |
| | qslA-R2:CGGGATCCGCCAAGCGCCAGGTCGGTCTCAT |
| | qslA-con-F:ATGACGAAGGATGAAAAGGAAAAG |
| | qslA-con-F:TCAACCGGAACGTCGATCGG |
| qslA complementation | qslA-For:GGAATTCCGCGCTCCAAGGCCGGGTTATTT |
| | qslA-Rev:CGGGATCCGCCAAGCGCCAGGTCGGTCTCAT |
| qslA expression | qslA-ATG:GGAATTCATGACGAAGGATGAAAAGGAAAAG |
| | qslA-TAA:CGGGATCCTCAACCGGAACGTCGATCGG |
| lasR deletion | lasR-F1: GGAATTCCAGCGTGGTGGTGTGCGAGATGTT |
| | lasR-R1:GTGCCCGACGACCCCTGAAGATGT |
| | lasR-F2:GGGGTCGTCGGGCACGCCTGGCGCGAGCATTACGAC |
| | lasR-R2:CGGGATCCCGCTACGCGGCGGGAGGTCAC |
| | lasR-con-F:TGGCCTTGGTTGACGGTTTTCTTG |
| lasR complementation | lasR-For:GGAATTCAGAGTGGGCTGACTGGACATCTTC |
| | lasR-Rev:CGGGATCCTTGTGCATCTCGCCCAGCAGTTTT |
| rhlR deletion | rhlR-F1: GGAATTCCTGCCGCCGGTACACCCCAAGTTC |
| | rhlR-R1:CAACCCTATCTGTTATGCCAGCACCG |
| | rhlR-F2:TAACAGATAGGGTTGTTCGACGCGCCGAACAAGACG |
| | rhlR-R2:CGGGATCCGTGCGCGAAACGGCTGACGACCT |
| rhlR complementation | rhlR-For:CCCAAGCTTGTTCGCGCCACCCCAGCAGGAT |
| | rhlR-Rev:GGAATTCTCAGATGAGGCCCAGCGCCGCG |
| mvfR deletion | mvfR -F1: GGAATTCCACGCGCCACCCAATAAAAGGAAT |
| | mvfR -R1:CGGCGGGATGGCGGTGTC |
| | mvfR -F2:ACCGCCATCCCGCCGCGCGCCGCACCAGAGTAGAG |
| | mvfR -R2:CCCAAGCTTGAGGCCGAGGAAACCCGCAAC |
| mvfR complementation | mvfR -For:GGAATTCCGCTCCACGACCGAACACGACA |
| | mvfR -Rev:CCCAAGCTTGGTCCGGGTGATGCGCAACGATAC |
| mvfR expression | mvfR-ATG:GGAATTC ATGGAACCCACCGAGCAGGCGCTT |
| | mvfR-TAG:CCCAAGCTTTGCAGTTCCATCCCGAGTCGATTCTC |
| |
| --- |
| |

## Slide 16
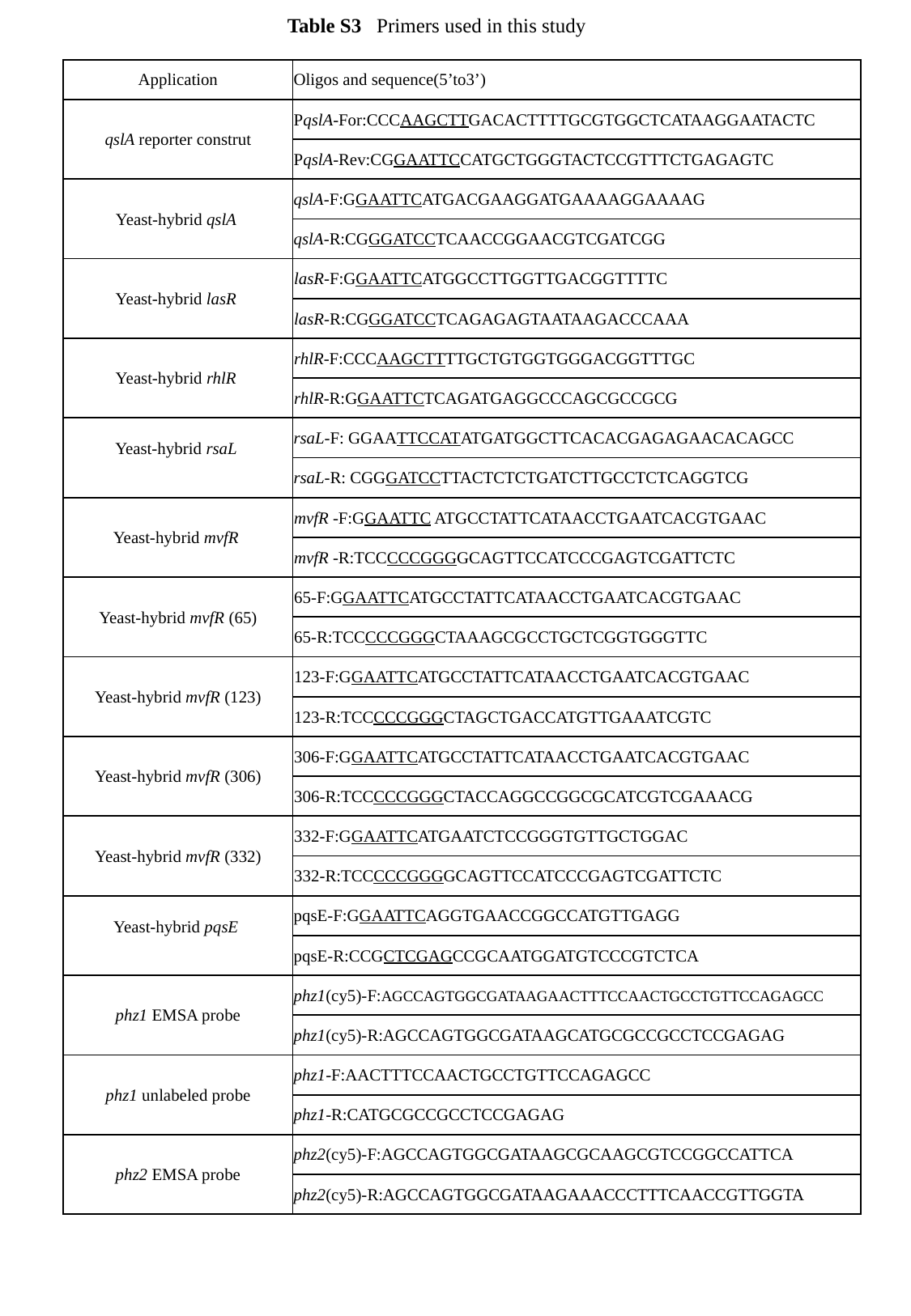

Table S3 Primers used in this study
| Application | Oligos and sequence(5’to3’) |
| --- | --- |
| qslA reporter construt | PqslA-For:CCCAAGCTTGACACTTTTGCGTGGCTCATAAGGAATACTC |
| | PqslA-Rev:CGGAATTCCATGCTGGGTACTCCGTTTCTGAGAGTC |
| Yeast-hybrid qslA | qslA-F:GGAATTCATGACGAAGGATGAAAAGGAAAAG |
| | qslA-R:CGGGATCCTCAACCGGAACGTCGATCGG |
| Yeast-hybrid lasR | lasR-F:GGAATTCATGGCCTTGGTTGACGGTTTTC |
| | lasR-R:CGGGATCCTCAGAGAGTAATAAGACCCAAA |
| Yeast-hybrid rhlR | rhlR-F:CCCAAGCTTTTGCTGTGGTGGGACGGTTTGC |
| | rhlR-R:GGAATTCTCAGATGAGGCCCAGCGCCGCG |
| Yeast-hybrid rsaL | rsaL-F: GGAATTCCATATGATGGCTTCACACGAGAGAACACAGCC |
| | rsaL-R: CGGGATCCTTACTCTCTGATCTTGCCTCTCAGGTCG |
| Yeast-hybrid mvfR | mvfR -F:GGAATTC ATGCCTATTCATAACCTGAATCACGTGAAC |
| | mvfR -R:TCCCCCGGGGCAGTTCCATCCCGAGTCGATTCTC |
| Yeast-hybrid mvfR (65) | 65-F:GGAATTCATGCCTATTCATAACCTGAATCACGTGAAC |
| | 65-R:TCCCCCGGGCTAAAGCGCCTGCTCGGTGGGTTC |
| Yeast-hybrid mvfR (123) | 123-F:GGAATTCATGCCTATTCATAACCTGAATCACGTGAAC |
| | 123-R:TCCCCCGGGCTAGCTGACCATGTTGAAATCGTC |
| Yeast-hybrid mvfR (306) | 306-F:GGAATTCATGCCTATTCATAACCTGAATCACGTGAAC |
| | 306-R:TCCCCCGGGCTACCAGGCCGGCGCATCGTCGAAACG |
| Yeast-hybrid mvfR (332) | 332-F:GGAATTCATGAATCTCCGGGTGTTGCTGGAC |
| | 332-R:TCCCCCGGGGCAGTTCCATCCCGAGTCGATTCTC |
| Yeast-hybrid pqsE | pqsE-F:GGAATTCAGGTGAACCGGCCATGTTGAGG |
| | pqsE-R:CCGCTCGAGCCGCAATGGATGTCCCGTCTCA |
| phz1 EMSA probe | phz1(cy5)-F:AGCCAGTGGCGATAAGAACTTTCCAACTGCCTGTTCCAGAGCC |
| | phz1(cy5)-R:AGCCAGTGGCGATAAGCATGCGCCGCCTCCGAGAG |
| phz1 unlabeled probe | phz1-F:AACTTTCCAACTGCCTGTTCCAGAGCC |
| | phz1-R:CATGCGCCGCCTCCGAGAG |
| phz2 EMSA probe | phz2(cy5)-F:AGCCAGTGGCGATAAGCGCAAGCGTCCGGCCATTCA |
| | phz2(cy5)-R:AGCCAGTGGCGATAAGAAACCCTTTCAACCGTTGGTA |

## Slide 17
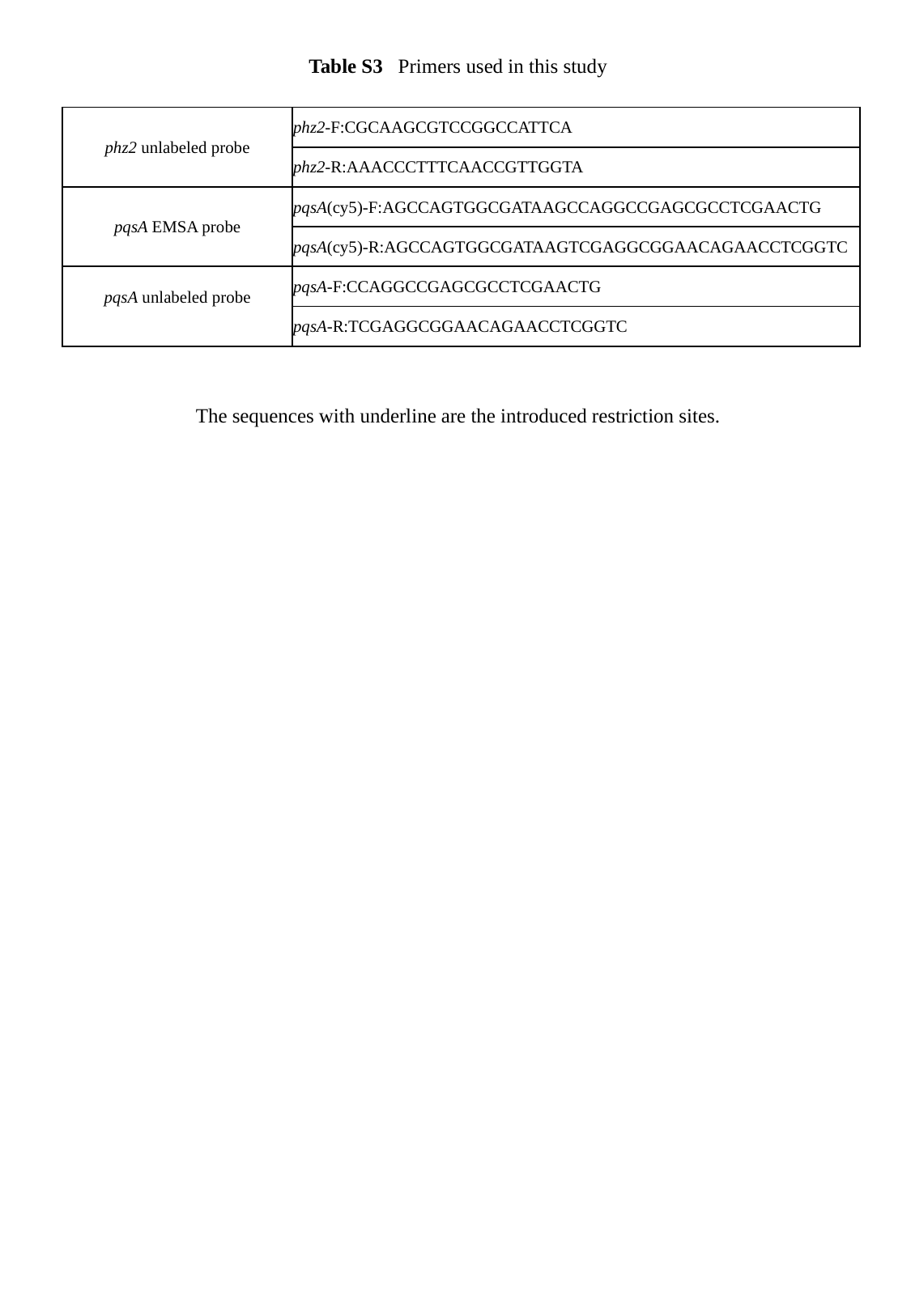

Table S3 Primers used in this study
| phz2 unlabeled probe | phz2-F:CGCAAGCGTCCGGCCATTCA |
| --- | --- |
| | phz2-R:AAACCCTTTCAACCGTTGGTA |
| pqsA EMSA probe | pqsA(cy5)-F:AGCCAGTGGCGATAAGCCAGGCCGAGCGCCTCGAACTG |
| | pqsA(cy5)-R:AGCCAGTGGCGATAAGTCGAGGCGGAACAGAACCTCGGTC |
| pqsA unlabeled probe | pqsA-F:CCAGGCCGAGCGCCTCGAACTG |
| | pqsA-R:TCGAGGCGGAACAGAACCTCGGTC |
The sequences with underline are the introduced restriction sites.
